# Healthcare contacts with self-harm during COVID-19: An e-cohort whole-population-based study using individual-level linked routine electronic health records in Wales, UK, 2016—March 2021

**DOI:** 10.1371/journal.pone.0266967

**Published:** 2022-04-27

**Authors:** M. DelPozo-Banos, S. C. Lee, Y. Friedmann, A. Akbari, F. Torabi, K. Lloyd, R. A. Lyons, A. John

**Affiliations:** 1 Swansea University Medical School, Wales, United Kingdom; 2 Population Data Science, Swansea University Medical School, Wales, United Kingdom; University of New South Wales, AUSTRALIA

## Abstract

**Introduction:**

Reduced rates of help seeking by those who self-harmed during the COVID-19 pandemic have been reported.

**Objectives:**

To understand changes in healthcare service contacts for self-harm during the COVID-19 pandemic across primary, emergency and secondary care.

**Methods:**

This retrospective cohort study used routine electronic healthcare data for Wales, United Kingdom, from 2016 to March 14, 2021. Population-based data from primary care, emergency departments and hospital admissions were linked at individual-level. All Welsh residents aged ≥10 years over the study period were included in the study. Primary, emergency and secondary care contacts with self-harm at any time between 2016 and March 14, 2021 were identified. Outcomes were counts, incidence, prevalence and proportion of self-harm contacts relative to all contacts in each and all settings, as well as the proportion of people contacting one or more settings with self-harm. Weekly trends were modelled using generalised estimated equations, with differences between 2020 (to March 2021) and comparison years 2016–2018 (to March 2017–2019) quantified using difference in differences, from which mean rate of odds ratios (μROR) across years was reported.

**Results:**

The study included 3,552,210 individuals over the study period. Self-harm contacts reduced across services in March and December 2020 compared to previous years. Primary care contacts with self-harm reduced disproportionately compared to non-self-harm contacts (μROR = 0.7, p<0.05), while their proportion increased in emergency departments during April 2020 (μROR = 1.3, p<0.05 in 2/3 comparison years) and hospital admissions during April-May 2020 (μROR = 1.2, p<0.05 in 2/3 comparison years). Despite this, those who self-harmed in April 2020 were more likely to be seen in primary care than other settings compared to previous years (μROR = 1.2, p<0.05). A lower proportion of those with self-harm contacts in emergency departments were subsequently admitted to hospital in December 2020 compared to previous years (μROR = 0.5, p<0.05).

**Conclusions:**

These findings suggest that those who self-harmed during the COVID-19 pandemic may have been less likely to seek help, and those who did so faced more stringent criteria for admission. Communications encouraging those who self-harm to seek help during pandemics may be beneficial. However, this needs to be supported by maintained provision of mental health services.

## Introduction

Early in the COVID-19 pandemic, concerns were raised about potential unintended consequences on peoples’ mental health of measures, such as ‘stay at home orders’, taken to curb the spread of the virus [1]. Some suggested increased rates of self-harm and suicidal behaviours in the population during the COVID-19 pandemic [2, 3], with some authors positively linking this with COVID-19 infection [4, 5]. However, while some studies on healthcare service utilisation for self-harm and suicidal thoughts/behaviours also described an increase during the first months of the pandemic (March-August 2020) in the number of presentations to emergency departments (EDs), psychiatric emergency services and trauma centres [6, 7], most reported a reduction compared to previous years [8]. In the United Kingdom (UK) and Ireland, primary care consultations and referrals to secondary care for self-harm fell by over 30% between March and April 2020, before returning to pre-pandemic levels by May-July 2020 [9, 10].

This decreased contact across services could reflect a real reduction in rates of self-harm (although community surveys do not indicate this is the case [5]) or reduced help-seeking. If the latter, there are concerns related to unmet need and missed opportunities for appropriate management of those who self-harm such as psychosocial assessments and evidence-based interventions [11]. However, many of these studies were based on single healthcare settings and/or specific sites and sub-populations, and mostly limited to Wave 1 of the pandemic. This hinders the generalisation of findings and the identification of the affected care pathways across settings necessary to understand and develop appropriate changes to policy and practice.

To address these limitations and better understand the effect of the COVID-19 pandemic on healthcare service contacts with self-harm, we used population-based routine data (Wales, UK) across healthcare settings (primary care, EDs and hospitals) during Waves 1 and 2 of the pandemic compared with similar periods in counterfactual years 2016–2019.

## Materials and methods

Ethical approval was granted by the Secure Anonymised Information Linkage (SAIL) Databank’s independent Information Governance Review Panel (project 0911), under SAIL Databank permissions for the analysis of anonymised linked data [12]. Only fully anonymised data sources were accessed for this study and therefore individual consent was not required. Results requested out of the SAIL gateway were independently reviewed by a SAIL Data Guardian to ensure compliance with information governance policies.

### Data

SAIL is a privacy-protecting trusted research environment which holds anonymised population-scale, individual-level linkable data pertaining to the population of Wales [13] (2020 mid-year estimate = 3.2 million individuals [14]). Health data in SAIL is provided by the National Health Service (NHS), which provides treatment free of charge to those lawfully entitled to be in the UK who usually live there, including refugees and asylum seekers with an active application or appeal. We used all data linked deterministically or probabilistically with linkage score ≥0.9 from the datasets in Table 1 [15].

**Table 1:** Data sources used in this analysis, updated frequently to support COVID-19 research.

Database	Description	Coverage at time of data extraction (28/04/2021)
Welsh Demographic Service	An administrative register of all individuals in Wales that use NHS services, containing anonymised demographics and GP practice registration history with anonymised residential data	Up to 25/4/2021
Office for National Statistics–Mortality register§	Death register of all deaths and causes in Wales, coded using ICD, version 10 codes, derived from information collected at registration of death. Daily and monthly extracts available.	Up to 28/3/2021
Consolidated Deaths Data Source§	Combination of death records from the Wales Demographic Service Dataset, Master Patient Index and ONS Deaths.	Up to 18/4/2021
Welsh Longitudinal General Practice	Primary care records with diagnoses, symptoms, investigations, prescribed medication, referrals, coded hospital contacts, and test results coded using Read Codes v2	80% (330/412) of all general practices in Wales up to 21/3/2021
Emergency Department Data Set†	Administrative and clinical information (general reason for attendance and attendance group to identify types of contacts) for all NHS Wales Accident and Emergency department attendances.	Up to 25/4/2021
Patient Episode Database for Wales‡	Clinical information (specialty and diagnoses) of all NHS Wales hospital admissions (inpatient and day cases)–diagnostic information coded using ICD-10 codes.	Up to 25/4/2021

ICD–International Classification of Diseases.

ONS–Office for National Statistics.

* Data have full Wales coverage throughout the study period unless otherwise shown.

§ User guide to mortality statistics—Office for National Statistics. [Online]. Available: https://www.ons.gov.uk/peoplepopulationandcommunity/birthsdeathsandmarriages/deaths/methodologies/userguidetomortalitystatisticsjuly2017.

† Digital Health and Care Wales (previously NHS Wales Informatics Service), Emergency Department Data Set structure. [Online]. Available: http://www.datadictionary.wales.nhs.uk/#!WordDocuments/datasetstructure4.htm.

‡ Digital Health and Care Wales (previously NHS Wales Informatics Service), Patient Episode Database for Wales Data Set structure. [Online]. Available: http://www.datadictionary.wales.nhs.uk/#!WordDocuments/datasetstructure.htm.

### Study population

Our study population included people living in Wales between January 1, 2016 and March 14, 2021, followed up from January 1, 2016, their 10^th^ birthday or the date they registered with a primary care provider (general practice; GP) using a Welsh address (whichever was latest); until March 14, 2021, death or the date they stopped being registered with a GP using a Welsh address (whichever was earliest). Intermittent follow-up periods were allowed (e.g. people moving in and out of Wales).

### Measures

We measured variables weekly as per the ISO week date standard (ISO-8601; www.iso.org/iso-8601-date-and-time-format.html) from January 4, 2016 (week 1 of 2016) to March 14, 2021 (week 10 of 2021). We defined Wave 1 of the pandemic from March 9, 2020 (week of the suspension of non-urgent NHS appointments [16]) to August 16, 2020; and Wave 2 from August 17, 2020 (week at which the number of COVID-19 infections started to rise again based on data in SAIL) to March 14, 2021. For each week, we considered the sub-population meeting the inclusion criteria on the Monday, and extracted demographic variables sex, age categories (10–24, >24 years), and level of deprivation. Specified age categories were selected to avoid small numbers. We identified deprivation level per individual using the Lower-layer Super Output Area of residence version 2011 (containing approximately 1500 individuals each) and the Welsh Index of Multiple Deprivation (WIMD) 2014 score, a composite measure that combines several different types of deprivation [17]. WIMD deprivation levels 1–5 (lowest-to-highest) were defined using national WIMD score quintiles as cut-offs.

We examined all contacts with GP, ED and hospital admissions independently. We defined ‘contact’ as a recorded entry in one of the data sources. In GP data, we excluded administrative codes and associated diagnoses such as ‘letter from ED’ but included telephone and face-to-face contacts [18]. For hospital admissions, we collapsed hospital transfers into a single continuous admission, propagated diagnoses across episodes within an admission and measured admissions and discharges.

We identified contacts with recorded self-harm, defined as ‘an intentional act of self-poisoning or self-injury, irrespective of the motivation or intent of the act’. We included all forms of intentional self-harm, from non-suicidal self-injury through to suicide attempts, with the exception of intentional alcohol poisoning or alcohol overdose. In keeping with previous published work in electronic health records [19, 20], we also included events of undetermined intent, as they are probable self-harm: hanging, strangulation and suffocation; rifle shotgun and larger firearm discharges and other and unspecified firearm discharges; and falling, jumping from a high place; events of overdose of drug, biological substances or medicament (without further specification); and poisoning and overdose of antidepressants (commonly implicated in suicide), without mention of intent. Events of poisoning and overdose of analgesics, although commonly implicated in suicidal behaviours as well, were not included as they are also commonly accidental. Identification was based on validated primary care Read codes, ED attendance type and diagnosis codes, and the International Classification of Diseases 10^th^ version codes for self-harm (X60-X84) and undetermined intent (Y10-Y34) for hospital admissions–full details and exhaustive code lists can be found in [19].

Linking across data sources, we measured number and proportion of self-harm contacts relative to all contacts in all settings combined and in each individual setting, as well as admissions from ED to hospital and hospital admissions with transfers to critical care. Admissions from ED to hospital were collapsed to avoid double counting episodes, but diagnoses were not propagated across settings. Thus, the number of GP, ED and hospital contacts separately do not necessarily add up to the number of contacts across all settings. These measures ascertained how self-harm contacts changed not only in absolute terms (raw counts), but also relative to non-self-harm contacts.

We measured the number of individuals with self-harm contacts and the proportion of them contacting one or more settings with self-harm. This included three overlapping sets (‘seen in GP’, ‘seen in ED’, and ‘seen in hospital admissions’) and seven non-overlapping sets (‘seen in GP only’; ‘seen in ED only’; ‘seen in GP and ED only’; etc) constituting a time-varying Venn diagram. The measures ascertained changes in the distribution of self-harm contacts across settings.

### Statistical analysis

We used SQL DB2 (www.ibm.com/analytics/db2) to interrogate data within the SAIL Databank and calculate counts, proportions, incidence and prevalence; Python (www.python.org) to represent the results; and R (www.r-project.org) and Stata version 16.1 (www.stata.com) for statistical analyses. The level of statistical significance was set at *p* = 0.05.

We compared changes in healthcare service utilisation between ‘before’ (December 30, 2019 to March 8, 2020 –start of COVID-19 Wave 1) and ‘during’ (March 9, 2020 to March 14, 2021 –non-urgent NHS appointments were suspended in Wales on March 13, 2020 [16]) COVID-19 periods to equivalent periods in pre-COVID-19 years 2016–2019. We used generalised estimating equations to model weekly trends of outcomes adjusting for demographic variables. We quantified differences between trends in 2020 (to March 2021) and 2016–2018 (to March 2017–2019) using the difference in difference (DiD) approach [21] to account for background fluctuations [22], and present ratio of rate ratios (RRRs) for counts and ratio of odds ratios (RORs) for proportions. For readability, in the text we report mean RRRs (μRRRs) and RORs (μRORs) across counterfactual years, for which p<0.05 in all years unless otherwise specified. Full results (per year) in S1–S8 Tables. Underlying linear trends were assessed using linear regression, comparing 2020 with years 2016–2019. Bonferroni adjustment was used to correct for multiple comparisons. We repeated these analyses stratifying by age-sex and WIMD deprivation quintile separately. Detailed model specifications, sensitivity analyses and robustness checks are described in S1–S3 Methods, as is the exact definition of incidence and prevalence.

## Results

**Fig 1:**
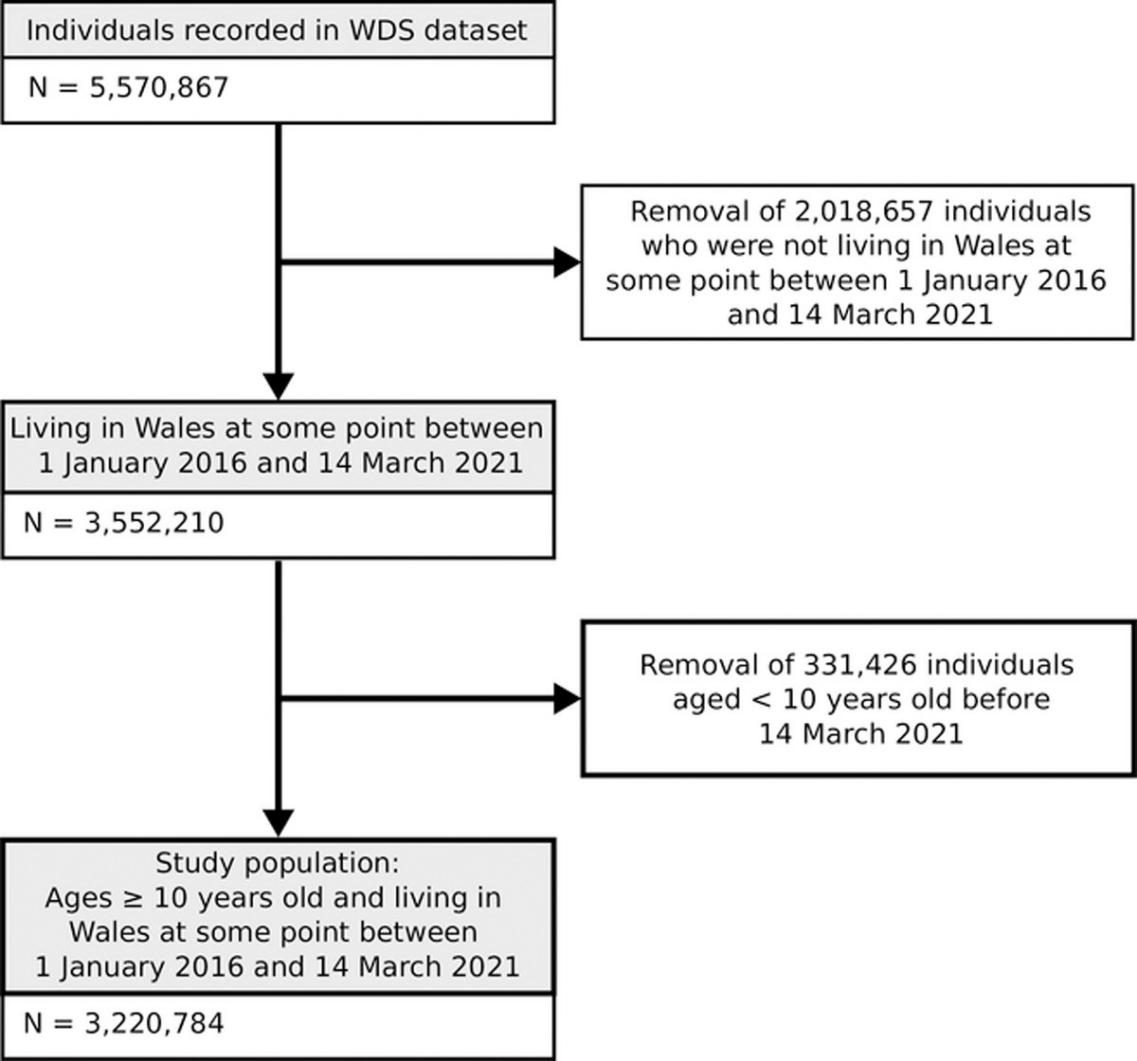
Study population flow diagram. Inclusion and exclusion decisions leading to the creation of the study population.

Of the 5,570,867 individuals recorded in SAIL, we identified 3,220,784 individuals meeting the inclusion criteria during the study period (Fig 1), of whom 1,770,973 (49.85%) were males, 190,919 (5.93%) turned 10 years old between January 1, 2016 and March 14, 2021, and 288,256 (8.95%) moved into Wales after January 1, 2016. The study population on January 4, 2016 (i.e., week 1) was 2,752,017, of whom 541,530 (19.68%) were 10–24 years old and 2,210,487 (80.32%) were >24 years old.

Between January 4, 2016 and March 8, 2020 we identified a weekly average of 365,297 GP contacts, 15,402 ED contacts and 16,523 hospital admissions for any reason. In each setting, from March 9, 2020 the number of weekly contacts was lower than in previous years–except during December 2020 and January 2021, when the number of GP contacts was higher than usual. The total number of weekly contacts was used as the denominator when calculating the proportion of contacts with self-harm (S1 Fig).

**Fig 2:**
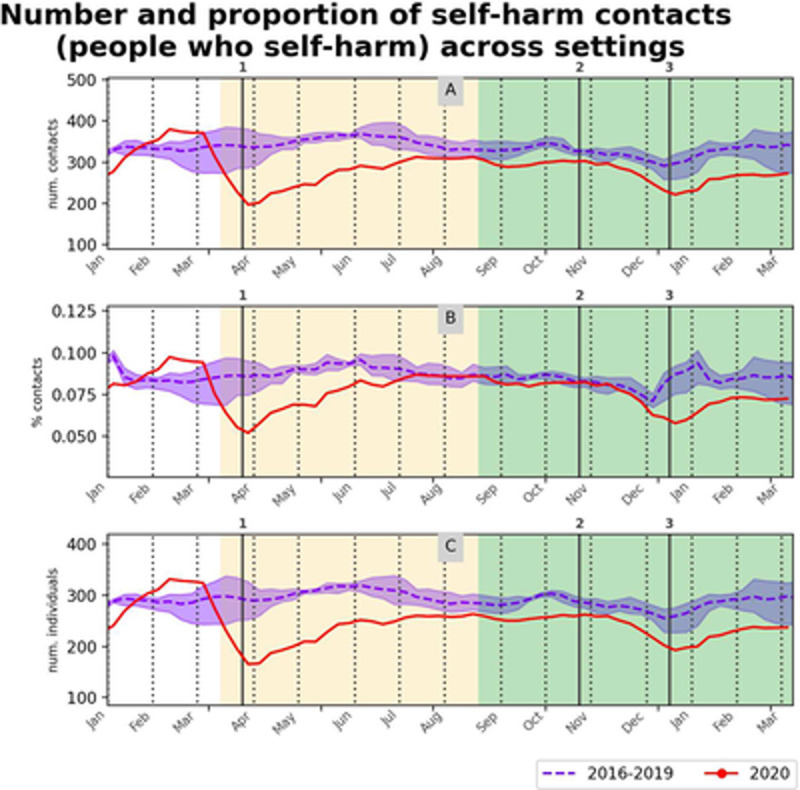
Healthcare service contacts with self-harm in any setting. (A) Count and (B) proportion of weekly contacts with self-harm in any setting (primary care, ED or hospital admissions). (C) Weekly count of individuals contacting any setting with self-harm. Solid red lines are 4-weeks rolling average of the weekly measurements for 2020. Blue dashed line and shaded area are average and min-max over the previous 4 years, 2016–2019. Changes in background shades correspond to before COVID-19, Wave 1 and Wave 2 periods respectively. Vertical lines are start stay-at-home measures during Wave 1 (1) and start of firebreak (2) and of stay-at-home (3) measures during Wave 2, in 2020.

Across settings, weekly counts of self-harm contacts reduced at the start of Wave 1 (μRRR = 0.6) and returned to pre-COVID-19 levels by July-August 2020 (μRRR = 0.9, p<0.05 for 1/3 counterfactual years). Wave 2 exhibited a second trough with a slightly less pronounced minimum than in Wave 1. This corresponded to a seasonal effect seen every Christmas but was amplified during 2020 compared to previous years (μRRR = 0.7). The weekly proportion of self-harm relative to all contacts and weekly number of individuals with self-harm contacts followed a similar trend. In all metrics (number and proportion of self-harm contacts and number of individuals with self-harm contacts), the downward trend was replaced by an upward trend shortly after the start of the stay-at-home measures in both waves (March 23 and December 16, 2020 respectively), and the drop during Wave 2 was slightly reduced compared to Wave 1. All these trends can be seen in Fig 2 (detailed results in S1 Table).

**Fig 3:**
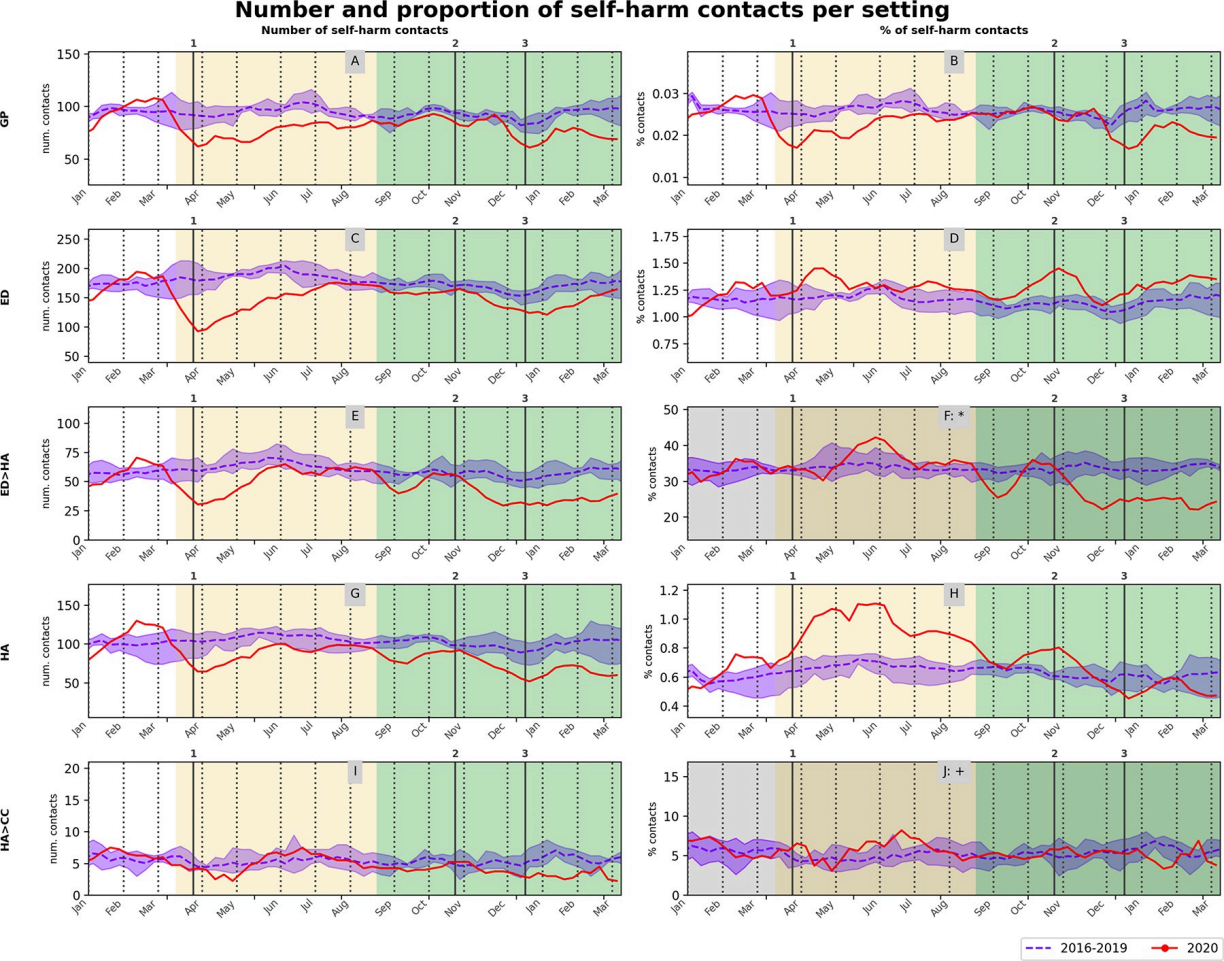
Healthcare service contacts with self-harm per setting. Count (left) and proportion (right) of weekly self-harm contacts in each setting–(A, B) primary care (GP), (C, D) ED, (E, F*) ED followed by hospital admissions (ED>HA), (G, H) hospital admissions (HA) and (I, J+) hospital admissions with a transfer to critical care (HA>CC). Solid red lines are 4-weeks rolling average of the weekly measurements for 2020. Blue dashed lines and shaded areas are average and min-max respectively over the previous 4 years, 2016–2019. Changes in background shades correspond to before COVID-19, Wave 1 and Wave 2 periods respectively. Vertical lines are start stay-at-home measures during Wave 1 (1) and start of firebreak (2) and of stay-at-home (3) measures during Wave 2, in 2020. Darker panels show proportion of ED contacts with self-harm that resulted in a hospital admission (F: *) and of hospital admissions with self-harm that resulted in a transfer to critical care (J: +).

The proportion of self-harm relative to all contacts in GP was lower in March-June 2020 (μROR = 0.7) and December 2020 (μROR = 0.7) compared to previous years. The proportion of self-harm relative to all contacts in ED peaked in April 2020 (μROR = 1.3, p<0.05 for 2/3 counterfactual years) and October-November 2020 (μROR = 1.3, p<0.05 for 2/3 counterfactual years) above pre-COVID-19 levels. The proportion of ED contacts with self-harm hospitalised followed a downward trend from June 2020 (monthly reduction of 1.9; 95% confidence interval (CI) [1.5, 2.3] percentage points (pp)). This trend flattened during the start of stay-at-home measures of Wave 2 at below pre-COVID-19 levels (μROR = 0.5), and there was no clear upward trend as seen in other settings. The proportion of self-harm admissions relative to all hospital admissions exhibited a sustained peak above pre-COVID-19 levels during Wave 1 (μROR = 1.2, p<0.05 for 2/3 counterfactual years), and a downward trend from June 2020 (monthly reduction of 0.06; 95%CI [0.07, 0.05] pp). The proportion of these admissions transferred to critical care remained constant during the study period. Weekly number and proportion of self-harm relative to all contacts per setting can be seen in Fig 3 (detailed results in S1 and S2 Tables).

**Fig 4:**
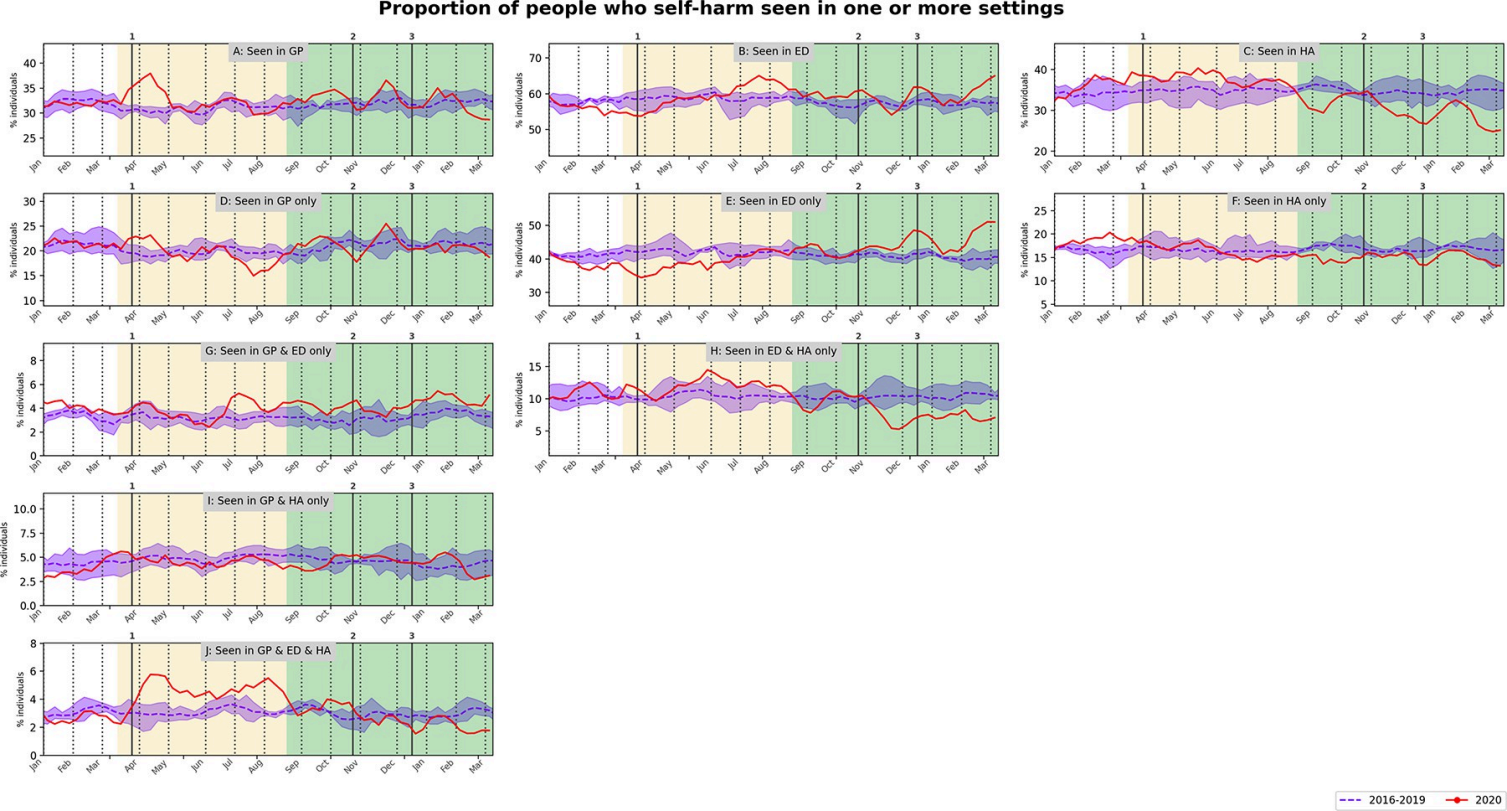
People in contact with one or more healthcare settings with self-harm. Weekly proportion of individuals with self-harm contacts seen in primary care (GP), ED and/or hospital admissions (HA). Solid red lines are 4-weeks rolling average of the weekly measurements for 2020. Blue dashed lines and shaded areas are average and min-max over the previous 4 years, 2016–2019. Panels A to C show overlapping sets. Panels D to J show non-overlapping sets. Changes in background shades correspond to before COVID-19, Wave 1 and Wave 2 periods respectively. Vertical lines are start stay-at-home measures during Wave 1 (1) and start of firebreak (2) and of stay-at-home (3) measures during Wave 2, in 2020.

In April 2020, compared to previous years people with self-harm contacts were more likely to be ‘seen in GP only’ (μROR = 1.2, p<0.05 for 1/3 counterfactual years) or ‘seen in all three settings’ (GP, ED and hospital admissions; μROR = 2.3), and less likely to be ‘seen in ED only‘ (μROR = 0.884, p<0.05 for 1/3 counterfactual years). During July-August 2020, increased ED contacts (μROR = 1.1) resulted in a decrease in the proportion ‘seen in GP only’ (μROR = 0.818, p <0.05 for 2/3 counterfactual years) and an increase in the proportion ‘seen in all three settings’ (μROR = 2.1). Going into Wave 2 and up to December 2020, fewer individuals with self-harm contacts were admitted to hospital (monthly reduction of 1 [0.3, 1.7] pp, p<0.05 for 2/4 counterfactual years), resulting in fewer individuals ‘seen in ED and hospital admissions only’ (monthly reduction of 1.2; 95%CI [0.6, 1.7] pp) and more ‘seen in ED only’ (monthly increase of 1; 95%CI [0.6, 1.3] pp). Towards the end of the study period (February 2021), the proportion of those with self-harm contacts ‘seen in ED only’ peaked above prior years (μROR = 1.3). Fig 4 shows how people with self-harm contacts distributed across settings (detailed results in S2 and S3 Tables).

The proportion of self-harm contacts due to burning increased in ED (μROR = 2.3 and 1.7 for Waves 1 and 2 respectively) and decreased in hospital admissions (μROR = 0.7 and 0.1 for Waves 1 and 2 respectively). The proportion of self-harm contacts due to hanging increased in Wave 1 for GP and hospital admissions (μROR = 2.0 and 1.4 respectively) but not in Wave 2 (μROR = 0.8 and 1.0 respectively). Self-harm contacts due to other methods showed no change during the COVID-19 pandemic or their numbers where too small to test (S4 Table).

Further analysis of incidence and prevalence of self-harm contacts showed virtually identical patterns for new and recurrent events across all and for each setting (S2 Fig).

### Sex, age and deprivation stratification

Both age groups (10–24 and >24) had similar changes in self-harm contacts, with a weak signal of a larger drop in counts during Wave 1 in those aged >24 years (μRRR = 0.8, p<0.05 for 1/3 counterfactual years). Males had slightly less number of contacts with self-harm during both Waves, while females had a more marked drop during Wave 1 but same as pre-COVID-19 levels during Wave 2 (μRRR = 1.2, p<0.05 for 2/3 counterfactual years). Looking at proportion of self-harm to all contacts, both males and females had similar patterns during Wave 1, but females had slightly higher proportion during Wave 2 (μROR = 1.1, p<0.05 for 2/3 counterfactual years). The peaks seen in the proportion of self-harm relative to all contacts in ED (despite a drop in counts) were largely the result of contacts in those aged 10–24 years (μROR = 1.7, p<0.05 for 3/3 counterfactual years). During Wave 1, the proportion of patients with self-harm contacts ‘seen in hospital only’ (μROR = 1.5 for both age and sex comparisons, p<0.05 for 1/3 counterfactual years) and ‘seen in ED and hospital admissions only’ (unable to test due to low counts) increased only in females aged 10–24 years. In Wave 2, the number of self-harm hospital admissions in females aged 10–24 years remained constant while it dropped for other sex-age groups (μRRR = 1.4 for both age and sex comparisons)—resulting in a complementary increase in the proportion of self-harm relative to all hospital admissions (μROR = 1.4 for both age and sex comparisons respectively) in this group. Meanwhile, the number of hospital admissions with self-harm in those aged >24 years exhibited a downward trend from June to December 2020, more so in males (monthly reduction 3.4; 95%CI [2.7, 4.2] contacts) than females (monthly reduction 2.1; 95%CI [0.8, 3.3] contacts, p<0.05 for 1/4 counterfactual years). We found no changes in the gradient across deprivation levels. Detailed results stratified by age-sex can be found in S3–S5 Figs and S2, S5 and S6 Tables, and stratified by WIMD deprivation levels in S6–S8 Figs and S7 and S8 Tables. Analysis of incidence and prevalence of self-harm contacts showed no further differences across sex, age and WIMD deprivation levels (S9 and S10 Figs).

## Discussion

To our knowledge, this is the first study to present a detailed picture of the effect the COVID-19 crisis had on self-harm contacts with primary care, ED and hospital admissions separately and in conjunction, using routinely collected data covering an entire population (Wales) for over five years, including the first 12 months of the pandemic. An initial reduction in the number of self-harm contacts at the start of the COVID-19 pandemic, followed by a recovery to pre-COVID-19 levels in the next 3–5 months has been reported across France [23], Italy [24], Ireland [10], Portugal [25], the UK [9], and the USA [26]. Our results extend this pattern to Wave 2 of the pandemic, showing that the minimum levels of contacts in Waves 1 and 2 coincided with the start of the stay-at-home restrictions, and that such patterns took place across each individual setting (GP, ED and hospital inpatients) and in both prevalence and incidence of self-harm contacts. We found (in Welsh data) no evidence of an additional trough during August-September 2020 previously reported in combined data for Northern-Ireland, Scotland and Wales [9], suggesting that such drop may have been localized in the other nations or may have been an artefact. In October 23, 2020 Wales entered into a short lock-down period (firebreak), shortly before Wave 2’s trough in the number of ED presentations and hospital admissions with self-harm started.

Restrictions were slowly phased out non-simultaneously across areas in Wales, hindering the specification of hard end dates [27]. In general, the “stay at home” message was replaced by a “stay local” one in June 1, 2020 for Wave 1; November 9, 2020 for the firebreak; and March 12, 2021 for Wave 2. The only notable change we found around these dates was the proportion of hospitalised ED presentations and the number of hospital admissions with self-harm plateauing at pre-COVID-19 levels in the first week of June 2020.

Despite data based on self-report community surveys suggesting an increased rate of suicidal and self-harming thoughts and behaviours [2, 3], we did not find a corresponding increase in raw counts, incidence or prevalence rates of self-harm contacts with healthcare services compared to previous years–similar results have been reported for suicide rates [28]. Fear of being infected with COVID-19 through contact with healthcare services, a desire to ‘protect’ overstretched healthcare services, the conversion of mental health facilities to treat COVID-19 patients, and/or to the change of services to non-face-to-face modalities may all have played a part in the drop in service utilisation in general, not only self-harm contacts. The reduction in service utilisation due to self-harm may reflect a lack of help-seeking in those not requiring physical ED care or hospital admission at these settings during the pandemic. However motivations and suicidal intent for self-harm fluctuates, and therefore it is important that all those who self-harm receive appropriate assessment and intervention [29].

At times during both waves, self-harm contacts constituted a higher-than-usual proportion of all ED contacts and hospital admissions–a finding similar to that reported for the first two months of the pandemic [26]. This may explain the perception by ED practitioners of an increase in self-harm. Conversely, self-harm contacts in primary care reduced disproportionately during both Waves compared to non-self-harm contacts. This is critical, since the proportion of those contacting GPs with self-harm peaked by 6 percentage points in April 2020, and during the study period 20% were ‘seen in GPs only’ (Fig 3). A UK survey study during Wave 1 reported people feeling least safe and confident attending Accident and Emergency departments compared to other types of hospital contacts, such as for essential surgery or clinical blood test [30]. This fear and a willingness to protect secondary healthcare provision may have resulted in patients preferentially contacting GPs during Wave 1 but not Wave 2. At the same time, while there were no restrictions on primary care utilisation, a rapid transformation to phone and video consultations may have initially prevented people from seeking help in relation to self-harm.

Population-level unmet need related to the fall in self-harm presentations has been suggested, with people unlikely to have received psychosocial assessments and interventions [11]. We found the proportion of ED contacts with self-harm subsequently hospitalized and of hospital admissions with self-harm transferred to critical care remained constant during Wave 1, while the former dropped during Wave 2. In keeping with the literature we also found some evidence that more lethal methods such as hanging increased during the pandemic, particularly during Wave 1 [23]. These results suggest more stringent criteria for hospitalisation following ED contacts with self-harm was potentially in operation. The fact that the situation, far from improving during Wave 2, seemed to worsen, as the reduction in the proportion of hospitalisations following ED contact remained low even after an increase in the number of ED contacts with self-harm, may be cause for concern.

Increasing rates of suicidal behaviours and suicide deaths have been previously linked to social and economic crises [31–33]. Our results pertain to healthcare service presentations and may not reflect true changes in the prevalence of self-harm, specially considering that service contacts were broadly affected by the pandemic (S1 Fig). Additionally, the analysis here includes the first year of the COVID-19 pandemic, when many of the health, social and economic support policies were still in place. As these are phased out and as the full consequences of the pandemic become apparent, trends of suicidal behaviours may change.

### Sex, age and deprivation stratification

Stratified results from other published studies are highly heterogeneous, perhaps due to the relatively small samples used–usually collected in a single hospital. A UK population-based study with data from combined primary and secondary care-treated episodes during Wave 1 found a most marked drop in younger ages and most deprived areas [9]. However, even though we found similar patterns in Waves 1 and 2, further analysis showed that the drop was proportionately smaller in young people (10–25 years) relative to non-self-harm contacts. Similarly, despite a larger drop of raw counts in more deprived groups, we found that the deprivation gradient was not affected during the pandemic.

Our results, in line with other studies using UK data [34, 35], showed that the drop in those aged 10–24 years during Wave 1, although larger in females, was proportionately similar in both sexes–others have reported increases in young females’ ED presentations with self-harm [36]. However, raw counts of presentations with self-harm rose to pre-pandemic levels from mid-July onwards (i.e., mostly Wave 2) in females only. This late female-only increase in children and young people may be related to worst mental health outcomes [37, 38] and higher number of mental health service contacts [39, 40] than males, and/or an extension of increases seen in incidence of self-harm in adolescent females before the pandemic [41]. Conversely, we found that fewer male adults where hospitalized with self-harm during Wave 2 compared to pre-COVID-19.

Teachers and other school staff are often at the front line of self-harm related disclosures and conversations with young people. Other studies have reported a relationship between self-harm and school absenteeism and exclusions [42, 43]. In our results, the number of self-harm presentations in females aged 10–24 years showed a stepped increase during July, following the re-opening of schools in Wales on 29 June 2020 –a similar increase was not seen in previous years. However, the same was not true for males, and the number of presentations for females seemed to be unaffected by further school closures (i.e., these mirrored pre-COVID-19 levels). These discrepancies may be explained by the inclusion of non-school ages 18–24 in our analysis. Unfortunately, small numbers prevented us from pursuing more detailed analyses (e.g., by splitting the population into school and further/higher education ages).

### Limitations

Results from this study should be interpreted in context. Limitations of the use of routinely collected data for research purposes have been reported elsewhere [44]. Our metric of deprivation was based on pre-pandemic data, and therefore does not include changes in income, employment and other socioeconomic factors that took place during the pandemic. Misclassification bias was reduced using validated definitions of contacts and self-harm code lists [18, 19]. Despite using population-based data, small numbers prevented us from running statistical tests on hospital admissions with critical care transfers and on some method and sex-age stratified analyses. When all the assumptions of the DiD approach used are met, causal inference may be drawn from our findings. However, such assumptions (i.e. a common trend across years, the correct specification of the functional form of trends and the non-existence of unmeasured confounding) may not be valid neither are they easily verified [21, 22]. For example, our data may be missing key socioeconomic factors [45], and our DiD analyses did not disentangle the effects of social/medical isolation from the pandemic [46], hence limiting any strong causal inferences.

## Conclusions

Fear of infection, stay at home orders and ‘protect the NHS’ may have discouraged those who self-harm from accessing healthcare services. More stringent criteria for admission following self-harm may have been employed. The fact that self-harm contacts in primary care were disproportionately affected may be a cause for concern, since 20% of those contacting with self-harm do so to primary care only. Not providing the support needed to those who self-harm will likely have negative implications for individuals and health services [11]. Thus, public communication campaigns encouraging those who self-harm in pandemic times to seek help from healthcare services may be beneficial, particularly for young women early on and adult men as the pandemic prolongs. However, this needs to be paired with maintained provision of mental health services and crisis care pathways to ensure that those who do seek help receive it.

## Supporting information

S1 File The RECORD statement–checklist of items, extended from the STROBE statement, that should be reported in observational studies using routinely collected health data. (PDF)

S1 Methods Statistical analysis–Incidence and prevalence of self-harm contacts. (PDF)

S2 Methods Statistical analysis–modelling for weekly time trends and contrast of model coefficients using Difference-in-difference (DiD) approach. (PDF)

S3 Methods Sensitivity analyses. (PDF)

S1 Fig Number of contacts with healthcare services. Weekly number of contacts (A) across all settings, (B) primary care (GP), (C) emergency departments (ED), and (D) hospital admissions (HA). Solid red lines are 4-weeks rolling average of the weekly measurements for 2020. Blue dashed line and shaded area are average and min-max over the previous 4 years, 2016–2019. Changes in background shades correspond to before COVID-19, Wave 1 and Wave 2 periods respectively. Vertical lines are start stay-at-home measures during Wave 1 (1) and start of firebreak (2) and of stay-at-home (3) measures during Wave 2, in 2020.
(PDF)

S2 Fig Incidence and prevalence of healthcare service contacts with self-harm in any and each setting. Weekly incidence (left) and prevalence (right) of self-harm contacts (A, B) across all settings, (C, D) primary care (GP), (E, F) emergency departments (ED), (G, H) ED followed by hospital admissions (ED>HA), (I, J) hospital admissions (HA) and (K, L) hospital admissions with a transfer to critical care (HA>CC). Solid red lines are 4-weeks rolling average of the weekly measurements for 2020. Blue dashed line and shaded area are average and min-max over the previous 4 years, 2016–2019. Changes in background shades correspond to before COVID-19, Wave 1 and Wave 2 periods respectively. Vertical lines are start stay-at-home measures during Wave 1 (1) and start of firebreak (2) and of stay-at-home (3) measures during Wave 2, in 2020.
(PDF)

S3 Fig Healthcare service contacts with self-harm in any setting stratified by sex-age groups. (A) Count and (C) proportion of weekly self-harm contacts in any setting (primary care, emergency departments or hospital admissions) stratified by sex-age groups. (C) Weekly count of individuals with a self-harm contact in any setting stratified by sex-age groups. Solid red lines are 4-weeks rolling average of the weekly measurements for 2020. Blue dashed lines are averages over the previous 4 years, 2016–2019. Changes in background shades correspond to before COVID-19, Wave 1 and Wave 2 periods respectively. Vertical lines are start stay-at-home measures during Wave 1 (1) and start of firebreak (2) and of stay-at-home (3) measures during Wave 2, in 2020.
(PDF)

S4 Fig Healthcare service contacts with self-harm in each setting stratified by sex-age groups. Count (left) and proportion (right) of weekly self-harm contacts in each setting stratified by sex-age groups–(A, B) primary care (GP), (C, D) emergency departments (ED), (E, F*) ED followed by hospital admissions (ED>HA), (G, H) hospital admissions (HA) and (I, J+) hospital admissions with a transfer to critical care (HA>CC). Solid red lines are 4-weeks rolling average of the weekly measurements for 2020. Blue dashed lines and shaded areas are average and min-max respectively over the previous 4 years, 2016–2019. Changes in background shades correspond to before COVID-19, Wave 1 and Wave 2 periods respectively. Vertical lines are start stay-at-home measures during Wave 1 (1) and start of firebreak (2) and of stay-at-home (3) measures during Wave 2, in 2020. Darker panels show proportion of ED presentation with self-harm that resulted in a hospital admission (F*) and of hospital admissions with self-harm that resulted in a transfer to critical care (J+).
(PDF)

S5 Fig People in contact with one or more healthcare settings with self-harm stratified by sex-age groups. Weekly proportion of individuals with self-harm contacts seen in primary care (GP), emergency departments (ED) and/or hospital admissions (HA) stratified by sex-age groups. Solid red lines are 4-weeks rolling average of the weekly measurements for 2020. Blue dashed lines and shaded areas are average and min-max over the previous 4 years, 2016–2019. Panels A to C show overlapping sets. Panels D to J show non-overlapping sets. Changes in background shades correspond to before COVID-19, Wave 1 and Wave 2 periods respectively. Vertical lines are start stay-at-home measures during Wave 1 (1) and start of firebreak (2) and of stay-at-home (3) measures during Wave 2, in 2020.
(PDF)

S6 Fig Healthcare service contacts with self-harm in any setting stratified by WIMD deprivation levels. (A) Count and (C) proportion of weekly self-harm contacts in any setting (primary care, emergency departments or hospital admissions) stratified by sex-age groups. (C) Weekly count of individuals with a self-harm contact in any setting stratified by WIMD deprivation levels. Solid red lines are 4-weeks rolling average of the weekly measurements for 2020. Blue dashed lines are averages over the previous 4 years, 2016–2019. Changes in background shades correspond to before COVID-19, Wave 1 and Wave 2 periods respectively. Vertical lines are start stay-at-home measures during Wave 1 (1) and start of firebreak (2) and of stay-at-home (3) measures during Wave 2, in 2020.
(PDF)

S7 Fig Healthcare service contacts with self-harm in each setting stratified by WIMD deprivation levels. Count (left) and proportion (right) of weekly self-harm contacts in each setting stratified by WIMD deprivation levels–(A, B) primary care (GP), (C, D) emergency departments (ED), (E, F*) ED followed by hospital admissions (ED>HA), (G, H) hospital admissions (HA) and (I, J+) hospital admissions with a transfer to critical care (HA>CC). Solid red lines are 4-weeks rolling average of the weekly measurements for 2020. Blue dashed lines and shaded areas are average and min-max respectively over the previous 4 years, 2016–2019. Changes in background shades correspond to before COVID-19, Wave 1 and Wave 2 periods respectively. Vertical lines are start stay-at-home measures during Wave 1 (1) and start of firebreak (2) and of stay-at-home (3) measures during Wave 2, in 2020. Darker panels show proportion of ED presentation with self-harm that resulted in a hospital admission (F*) and of hospital admissions with self-harm that resulted in a transfer to critical care (J+).
(PDF)

S8 Fig People in contact with one or more healthcare settings with self-harm stratified by WIMD deprivation levels. Weekly proportion of individuals with self-harm contacts seen in primary care (GP), emergency departments (ED) and/or hospital admissions (HA) stratified by WIMD deprivation levels. Solid red lines are 4-weeks rolling average of the weekly measurements for 2020. Blue dashed lines and shaded areas are average and min-max over the previous 4 years, 2016–2019. Panels A to C show overlapping sets. Panels D to J show non-overlapping sets. Changes in background shades correspond to before COVID-19, Wave 1 and Wave 2 periods respectively. Vertical lines are start stay-at-home measures during Wave 1 (1) and start of firebreak (2) and of stay-at-home (3) measures during Wave 2, in 2020.
(PDF)

S9 Fig Incidence and prevalence of healthcare service contacts with self-harm in any and each setting stratified by sex-age groups. Weekly incidence (left) and prevalence (right) of self-harm contacts (A, B) across all settings, (C, D) primary care (GP), (E, F) emergency departments (ED), (G, H) ED followed by hospital admissions (ED>HA), (I, J) hospital admissions (HA) and (K, L) hospital admissions with a transfer to critical care (HA>CC) stratified by sex-age groups. Solid red lines are 4-weeks rolling average of the weekly measurements for 2020. Blue dashed line and shaded area are average and min-max over the previous 4 years, 2016–2019. Changes in background shades correspond to before COVID-19, Wave 1 and Wave 2 periods respectively. Vertical lines are start stay-at-home measures during Wave 1 (1) and start of firebreak (2) and of stay-at-home (3) measures during Wave 2, in 2020.
(PDF)

S10 Fig Incidence and prevalence of healthcare service contacts with self-harm in any and each setting stratified by WIMD deprivation levels. Weekly incidence (left) and prevalence (right) of self-harm contacts (A, B) across all settings, (C, D) primary care (GP), (E, F) emergency departments (ED), (G, H) ED followed by hospital admissions (ED>HA), (I, J) hospital admissions (HA) and (K, L) hospital admissions with a transfer to critical care (HA>CC) stratified by WIMD deprivation levels. Solid red lines are 4-weeks rolling average of the weekly measurements for 2020. Blue dashed line and shaded area are average and min-max over the previous 4 years, 2016–2019. Changes in background shades correspond to before COVID-19, Wave 1 and Wave 2 periods respectively. Vertical lines are start stay-at-home measures during Wave 1 (1) and start of firebreak (2) and of stay-at-home (3) measures during Wave 2, in 2020.
(PDF)

S1 Table RORs/RRRs of healthcare service contacts with self-harm in any and each setting. Summary of RORs and RRRs comparing weekly change in self-harm contacts between reference and target COVID-19 periods to the respective changes in previous years across settings (Any), per setting (primary care, GP; emergency departments (ED); and hospital admissions, HA) and for ED presentations with subsequent hospitalisation (ED to HA).
(PDF)

S2 Table Linear trends for number and proportion of self-harm contacts. Results of linear trends of weekly number and proportion of self-harm contacts and weekly proportion of people with self-harm contacts for primary care (GP), emergency departments (ED) and hospital admissions (HA).
(PDF)

S3 Table RORs of people in contact with one or more healthcare settings with self-harm. Summary of RORs comparing change in proportion of people who self-harmed and were in contact with primary care (GP), emergency departments (ED) and/or hospital admissions (HA) between reference and target periods to the respective changes in previous years.
(PDF)

S4 Table RORs/RRRs of healthcare service contacts with self-harm in each setting stratified by method. Summary of RRR/RORs based on the difference-in-difference (DiD) approach comparing changes in number and proportion of self-harm contacts per method across primary care (GP), emergency departments (ED) and hospital admissions (HA) between reference and target periods to the respective changes in previous years (as counterfactual).
(PDF)

S5 Table RORs/RRRs of healthcare service contacts with self-harm in any and each setting stratified by sex and age. Summary of RORs and RRRs for self-harm contacts stratified by age and sex for primary care (GP), emergency departments (ED) and hospital admissions (HA).
(PDF)

S6 Table RORs of people in contact with one or more healthcare settings with self-harm stratified by sex and age. Summary of RORs comparing change in proportion of people who self-harm and are in contact with primary care (GP), emergency departments (ED) and/or hospital admissions (HA) between reference and target periods to the respective changes in previous years stratified by age and sex.
(PDF)

S7 Table RORs/RRRs of healthcare service contacts with self-harm in any and each setting stratified by WIMD deprivation level. Summary of RORs and RRRs for change in gradient of self-harm contacts by WIMD quintile for all settings (Any), primary care (GP), emergency departments (ED) and hospital admissions (HA), as well as for ED presentations with subsequent hospitalisation (ED to HA).
(PDF)

S8 Table RORs of people in contact with one or more healthcare settings with self-harm stratified by WIMD deprivation level. Summary of RORs comparing change in proportion of people who self-harmed and were in contract with primary care (GP), emergency departments (ED) and/or hospital admissions (HA) between reference and target periods to the respective changes in previous years stratified by WIMD deprivation level.
(PDF)
