# Supplementary material for: Healthcare contacts with self-harm during COVID-19: An e-cohort whole-population-based study using individual-level linked routine electronic health records in Wales, UK, 2016—March 2021
Source: PLoS One. 2022 Apr 27;17(4):e0266967. doi: 10.1371/journal.pone.0266967 (PMC9045644; doi:10.1371/journal.pone.0266967)
Supplement: S1 Methods — (PDF) [file pone.0266967.s002.pdf]

Healthcare contacts with self-harm during COVID-19: an e-cohort whole-population-based study using individual-level linked routine electronic health records in Wales, UK, 2016 – March 2021

Marcos DelPozo-Banos, Sze Chim Lee, Yasmin Friedmann, Ashley Akbari, Fatemeh Torabi, Keith Lloyd, Ronan A Lyons, Ann John

S1 Methods. Statistical Analysis – Incidence and prevalence of self-harm contacts

For each week, we measured incidence and prevalence of self-harm contacts. Incidence contact was defined as the first contact in a 12-month period with data available, thus using 2015 data for the computation of 2016 incidences. During incidence and prevalence computation, only periods of known Welsh residency were considered. We measured ratio of rate ratios (RRRs) (and 95% confidence intervals, CIs) of 2020-March 2021 compared to each counterfactual period 2016-2019. Bonferroni adjustment was used to correct for multiple comparisons. Results can be found in S1 Fig., S9 Fig. and S10 Fig.
