# Supplementary material for: Healthcare contacts with self-harm during COVID-19: An e-cohort whole-population-based study using individual-level linked routine electronic health records in Wales, UK, 2016—March 2021
Source: PLoS One. 2022 Apr 27;17(4):e0266967. doi: 10.1371/journal.pone.0266967 (PMC9045644; doi:10.1371/journal.pone.0266967)
Supplement: S2 Methods — (PDF) [file pone.0266967.s003.pdf]

Healthcare contacts with self-harm during COVID-19: an e-cohort whole-population-based study using individual-level linked routine electronic health records in Wales, UK, 2016 – March 2021

Marcos DelPozo-Banos, Sze Chim Lee, Yasmin Friedmann, Ashley Akbari, Fatemeh Torabi, Keith Lloyd, Ronan A Lyons, Ann John

S2 Methods. Statistical Analysis – modelling for weekly time trends and contrast of model coefficients using Difference-in-difference (DiD) approach

We modelled weekly time trends via generalised estimating equations (GEE) using robust variance for parameter estimation (Agresti, 2007). We adopted the binomial distribution with logit link function for proportion outcomes and Poisson distribution with log link function for prevalence and incidence outcomes. The exchangeable within-subject correlation structure was chosen to circumvent correlation of outcomes over time based on the quasilikelihood under the independence model criterion as described previously (Cui & Qian, 2007; Pan, 2002). In all models, time was the key independent variable and as an indicator variable to estimate variation of outcomes each week within our observation period. We only included the weeks from the beginning of each year to the week corresponding to the end of the observation period in the modelling. Sex and age groups were represented by categorical variables and by WIMD quintile (1-5) as ranked categories. Only for modelling, we re-grouped age group as a two-level categorical variable (10-24 vs. >24 years) to circumvent a non-convergence issue due to small sample size in age group >64 years. We included three time-related 2nd order interaction terms in four separate models, namely, time-by-sex, time-by-age and time-by-WIMD to disentangle overall and time-varying differential effects of age, sex and area deprivation on self-harm outcomes.

We reported ratios (and 95% confidence intervals, CIs) of the odds/rate ratios of 2020/2021 compared to each counterfactual period 2016-2018/2019, i.e., ratio of odds ratios (RORs) for proportion and ratios of rate ratios (RRRs) for prevalence and incidence. RRRs/RORs different from one reflect a difference in 2020/2021 trend compared to the previous four years. Whether RRRs/RORs >1 or <1 depends on outcome trends. Arithmetic means of the model coefficients were used for periods spanning more than one week (e.g., week 1 to 10). Comparisons across age groups, sex and deprivation level were also performed (i.e., triple differences) by examining whether the ratios of RRRs and RORs between subgroups are significantly different from unity. All ratios were compiled using ‘contrast’ command in Stata after modelling and Bonferroni adjustment was used to correct for multiple comparisons. We repeated these analyses stratifying by age, sex and WIMD quintile separately.

References

Agresti A (2007) *An Introduction to Categorical Data Analysis*. 2nd edn. Hoboken, NJ: John Wiley & Sons Inc.

Cui J, Qian G (2007) Selection of Working Correlation Structure and Best Model in GEE Analyses of Longitudinal Data, *Communications in Statistics - Simulation and Computation* **36**(5), 987–996. doi: 10.1080/03610910701539617.

Pan W (2002) Goodness-of-fit Tests for GEE with Correlated Binary Data, *Scandinavian Journal of Statistics* **29**(1), 101–110. doi: https://doi.org/10.1111/1467-9469.00091.
