# Supplementary material for: Healthcare contacts with self-harm during COVID-19: An e-cohort whole-population-based study using individual-level linked routine electronic health records in Wales, UK, 2016—March 2021
Source: PLoS One. 2022 Apr 27;17(4):e0266967. doi: 10.1371/journal.pone.0266967 (PMC9045644; doi:10.1371/journal.pone.0266967)
Supplement: S3 Methods — (PDF) [file pone.0266967.s004.pdf]

Healthcare contacts with self-harm during COVID-19: an e-cohort whole-population-based study using individual-level linked routine electronic health records in Wales, UK, 2016 – March 2021

Marcos DelPozo-Banos, Sze Chim Lee, Yasmin Friedmann, Ashley Akbari, Fatemeh Torabi, Keith Lloyd, Ronan A Lyons, Ann John

S3 Methods. Sensitivity analyses

To ascertain the effect data coverage may have had in our results, we replicated the main analysis (i.e., without stratification) using for each week the sub-population registered with a GP providing data to SAIL on that Monday. These results were similar to and yielded the same conclusions than the main results presented in the text.
