# Supplementary material for: Healthcare contacts with self-harm during COVID-19: An e-cohort whole-population-based study using individual-level linked routine electronic health records in Wales, UK, 2016—March 2021
Source: PLoS One. 2022 Apr 27;17(4):e0266967. doi: 10.1371/journal.pone.0266967 (PMC9045644; doi:10.1371/journal.pone.0266967)
Supplement: S6 Fig — (A) Count and (C) proportion of weekly self-harm contacts in any setting (primary care, emergency departments or hospital admissions) stratified by sex-age groups. (C) Weekly count of individuals with a self-harm contact in any setting stratified by WIMD deprivation levels. Solid red lines are 4-weeks rolling average of the weekly measurements for 2020. Blue dashed lines are averages over the previous 4 years, 2016–2019. Changes in background shades correspond to before COVID-19, Wave 1 and Wave 2 periods respectively. Vertical lines are start stay-at-home measures during Wave 1 (1) and start of firebreak (2) and of stay-at-home (3) measures during Wave 2, in 2020. (PDF) [file pone.0266967.s010.pdf]

Healthcare contacts with self-harm during COVID-19: an e-cohort whole-population-based study using individual-level linked routine electronic health records in Wales, UK, 2016 – March 2021

Marcos DelPozo-Banos, Sze Chim Lee, Yasmin Friedmann, Ashley Akbari, Fatemeh Torabi, Keith Lloyd, Ronan A Lyons, Ann John

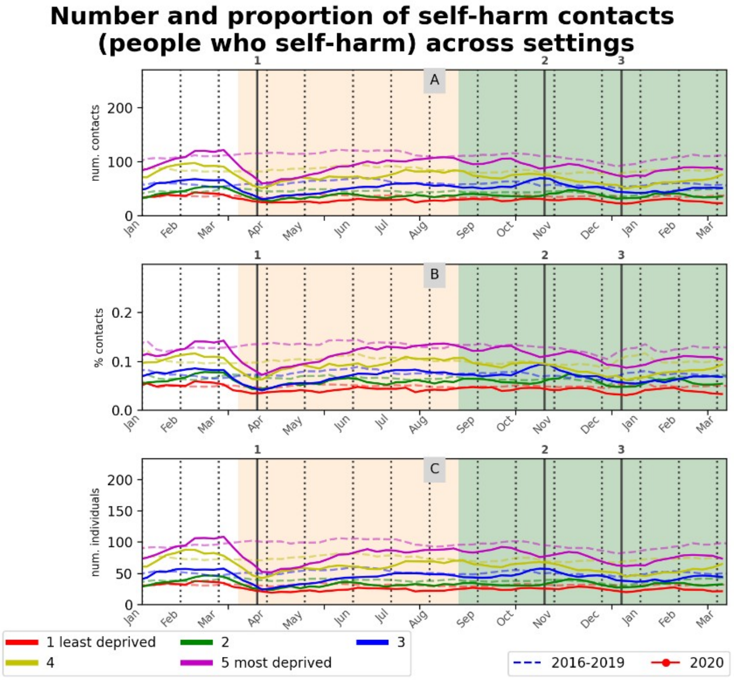

S6 Fig. Healthcare service contacts with self-harm in any setting stratified by WIMD deprivation levels. (A) Count and (C) proportion of weekly self-harm contacts in any setting (primary care, emergency departments or hospital admissions) stratified by sex-age groups. (C) Weekly count of individuals with a self-harm contact in any setting stratified by WIMD deprivation levels. Solid red lines are 4-weeks rolling average of the weekly measurements for 2020. Blue dashed lines are averages over the previous 4 years, 2016-2019. Changes in background shades correspond to before COVID-19, Wave 1 and Wave 2 periods respectively. Vertical lines are start stay-at-home measures during Wave 1 (1) and start of firebreak (2) and of stay-at-home (3) measures during Wave 2, in 2020.
