# Supplementary material for: Healthcare contacts with self-harm during COVID-19: An e-cohort whole-population-based study using individual-level linked routine electronic health records in Wales, UK, 2016—March 2021
Source: PLoS One. 2022 Apr 27;17(4):e0266967. doi: 10.1371/journal.pone.0266967 (PMC9045644; doi:10.1371/journal.pone.0266967)
Supplement: S7 Fig — Count (left) and proportion (right) of weekly self-harm contacts in each setting stratified by WIMD deprivation levels–(A, B) primary care (GP), (C, D) emergency departments (ED), (E, F*) ED followed by hospital admissions (ED>HA), (G, H) hospital admissions (HA) and (I, J+) hospital admissions with a transfer to critical care (HA>CC). Solid red lines are 4-weeks rolling average of the weekly measurements for 2020. Blue dashed lines and shaded areas are average and min-max respectively over the previous 4 years, 2016–2019. Changes in background shades correspond to before COVID-19, Wave 1 and Wave 2 periods respectively. Vertical lines are start stay-at-home measures during Wave 1 (1) and start of firebreak (2) and of stay-at-home (3) measures during Wave 2, in 2020. Darker panels show proportion of ED presentation with self-harm that resulted in a hospital admission (F*) and of hospital admissions with self-harm that resulted in a transfer to critical care (J+). (PDF) [file pone.0266967.s011.pdf]

Healthcare contacts with self-harm during COVID-19: an e-cohort whole-population-based study using individual-level linked routine electronic health records in Wales, UK, 2016 – March 2021

Marcos DelPozo-Banos, Sze Chim Lee, Yasmin Friedmann, Ashley Akbari, Fatemeh Torabi, Keith Lloyd, Ronan A Lyons, Ann John

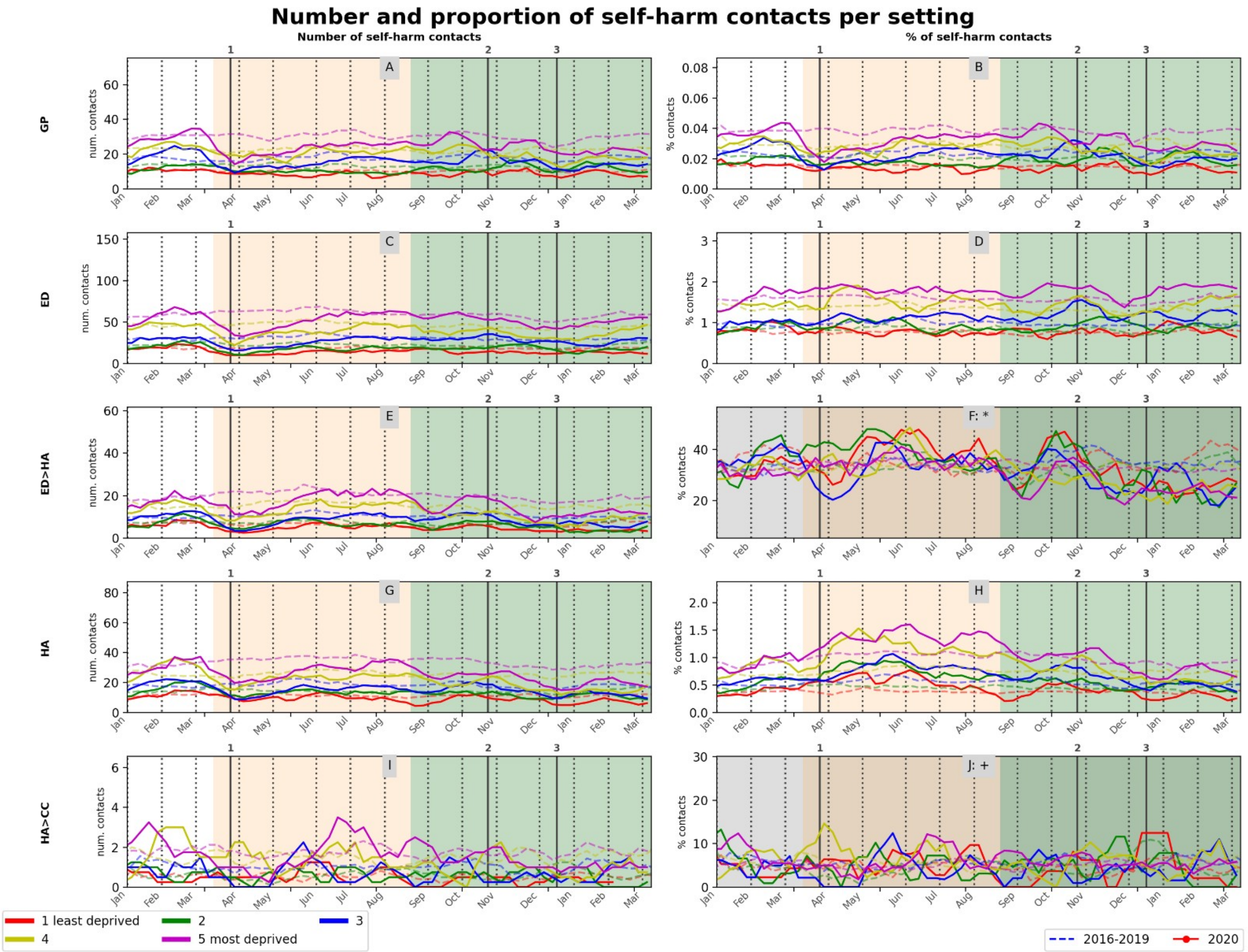

S7 Fig. Healthcare service contacts with self-harm in each setting stratified by WIMD deprivation levels. Count (left) and proportion (right) of weekly self-harm contacts in each setting stratified by WIMD deprivation levels – (A, B) primary care (GP), (C, D) emergency departments (ED), (E, F*) ED followed by hospital admissions (ED>HA), (G, H) hospital admissions (HA) and (I, J+) hospital admissions with a transfer to critical care (HA>CC). Solid red lines are 4-weeks rolling average of the weekly measurements for 2020. Blue dashed lines and shaded areas are average and min-max respectively over the previous 4 years, 2016-2019. Changes in background shades correspond to before COVID-19, Wave 1 and Wave 2 periods respectively. Vertical lines are start stay-at-home measures during Wave 1 (1) and start of firebreak (2) and of stay-at-home (3) measures during Wave 2, in 2020. Darker panels show proportion of ED presentation with self-harm that resulted in a hospital admission (F*) and of hospital admissions with self-harm that resulted in a transfer to critical care (J+).
