# Supplementary material for: Healthcare contacts with self-harm during COVID-19: An e-cohort whole-population-based study using individual-level linked routine electronic health records in Wales, UK, 2016—March 2021
Source: PLoS One. 2022 Apr 27;17(4):e0266967. doi: 10.1371/journal.pone.0266967 (PMC9045644; doi:10.1371/journal.pone.0266967)
Supplement: S9 Fig — Weekly incidence (left) and prevalence (right) of self-harm contacts (A, B) across all settings, (C, D) primary care (GP), (E, F) emergency departments (ED), (G, H) ED followed by hospital admissions (ED>HA), (I, J) hospital admissions (HA) and (K, L) hospital admissions with a transfer to critical care (HA>CC) stratified by sex-age groups. Solid red lines are 4-weeks rolling average of the weekly measurements for 2020. Blue dashed line and shaded area are average and min-max over the previous 4 years, 2016–2019. Changes in background shades correspond to before COVID-19, Wave 1 and Wave 2 periods respectively. Vertical lines are start stay-at-home measures during Wave 1 (1) and start of firebreak (2) and of stay-at-home (3) measures during Wave 2, in 2020. (PDF) [file pone.0266967.s013.pdf]

Healthcare contacts with self-harm during COVID-19: an e-cohort whole-population-based study using individual-level linked routine electronic health records in Wales, UK, 2016 – March 2021

Marcos DelPozo-Banos, Sze Chim Lee, Yasmin Friedmann, Ashley Akbari, Fatemeh Torabi, Keith Lloyd, Ronan A Lyons, Ann John

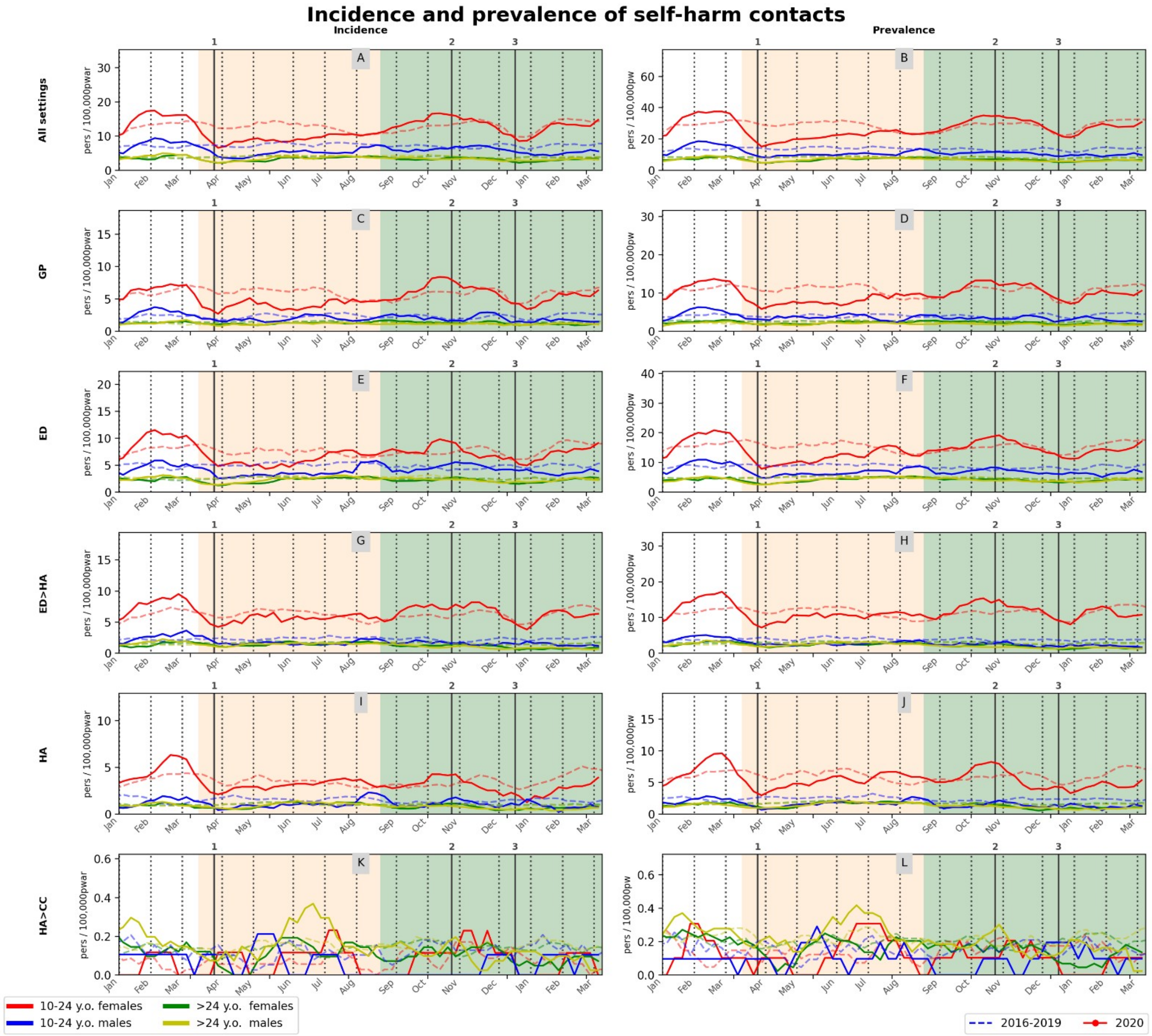

S9 Fig. Incidence and prevalence of healthcare service contacts with self-harm in any and each setting stratified by sex-age groups. Weekly incidence (left) and prevalence (right) of self-harm contacts (A, B) across all settings, (C, D) primary care (GP), (E, F) emergency departments (ED), (G, H) ED followed by hospital admissions (ED>HA), (I, J) hospital admissions (HA) and (K, L) hospital admissions with a transfer to critical care (HA>CC) stratified by sex-age groups. Solid red lines are 4-weeks rolling average of the weekly measurements for 2020. Blue dashed line and shaded area are average and min-max over the previous 4 years, 2016-2019. Changes in background shades correspond to before COVID-19, Wave 1 and Wave 2 periods respectively. Vertical lines are start stay-at-home measures during Wave 1 (1) and start of firebreak (2) and of stay-at-home (3) measures during Wave 2, in 2020.
