# Supplementary material for: Healthcare contacts with self-harm during COVID-19: An e-cohort whole-population-based study using individual-level linked routine electronic health records in Wales, UK, 2016—March 2021
Source: PLoS One. 2022 Apr 27;17(4):e0266967. doi: 10.1371/journal.pone.0266967 (PMC9045644; doi:10.1371/journal.pone.0266967)
Supplement: S1 Table — Summary of RORs and RRRs comparing weekly change in self-harm contacts between reference and target COVID-19 periods to the respective changes in previous years across settings (Any), per setting (primary care, GP; emergency departments (ED); and hospital admissions, HA) and for ED presentations with subsequent hospitalisation (ED to HA). (PDF) [file pone.0266967.s015.pdf]

Healthcare contacts with self-harm during COVID-19: an e-cohort whole-population-based study using individual-level linked routine electronic health records in Wales, UK, 2016 – March 2021

Marcos DelPozo-Banos, Sze Chim Lee, Yasmin Friedmann, Ashley Akbari, Fatemeh Torabi, Keith Lloyd, Ronan A Lyons, Ann John

S1 Table. RORs/RRRs of healthcare service contacts with self-harm in any and each setting. Summary of RORs and RRRs comparing weekly change in self-harm contacts between reference and target COVID-19 periods to the respective changes in previous years across settings (Any), per setting (primary care, GP; emergency departments (ED); and hospital admissions, HA) and for ED presentations with subsequent hospitalisation (ED to HA).

Setting	Outcome	Reference period[a]		Target period[a]		Year as counterfactual	RRR/ROR[b]	95% CI	*p*-value	*p*-value*
Any	numbers	week 1-10	30/12/2019	week 12-14	16/03/2020	2016-2017	0.576	(0.510, 0.652)	<0.001	**<0.001**
			to		to	2017-2018	0.576	(0.509, 0.652)	<0.001	**<0.001**
			08/03/2020		05/04/2020	2018-2019	0.600	(0.529, 0.680)	<0.001	**<0.001**
Any	numbers	week 1-10	30/12/2019	week 30-33	20/07/2020	2016-2017	0.979	(0.886, 1.082)	0.683	>0.999
			to		to	2017-2018	0.914	(0.828, 1.009)	0.074	0.223
			08/03/2020		16/08/2020	2018-2019	0.788	(0.714, 0.869)	<0.001	**<0.001**
Any	numbers	week 1-10	30/12/2019	week 50-53	07/12/2020	2016-2017	0.730	(0.654, 0.816)	<0.001	**<0.001**
			to		to	2017-2018	0.649	(0.582, 0.723)	<0.001	**<0.001**
			08/03/2020		03/01/2021	2018-2019	0.622	(0.557, 0.694)	<0.001	**<0.001**
Any	proportion	week 1-10	30/12/2019	week 12-14	16/03/2020	2016-2017	0.449	(0.446, 0.559)	<0.001	**<0.001**
			to		to	2017-2018	0.553	(0.494, 0.620)	<0.001	**<0.001**
			08/03/2020		05/04/2020	2018-2019	0.538	(0.478, 0.605)	<0.001	**<0.001**
Any	proportion	week 1-10	30/12/2019	week 30-33	20/07/2020	2016-2017	1.025	(0.936, 1.124)	0.590	>0.999
			to		to	2017-2018	0.925	(0.879, 1.054)	0.407	>0.999
			08/03/2020		16/08/2020	2018-2019	0.829	(0.757, 0.908)	<0.001	**<0.001**
Any	proportion	week 1-10	30/12/2019	week 50-53	07/12/2020	2016-2017	0.763	(0.692, 0.842)	<0.001	**<0.001**
			to		to	2017-2018	0.734	(0.666, 0.810)	<0.001	**<0.001**
			08/03/2020		03/01/2021	2018-2019	0.681	(0.617, 0.753)	<0.001	**<0.001**
GP	numbers	week 1-10	30/12/2019	week 12-14	16/03/2020	2016-2017	0.665	(0.535, 0.826)	<0.001	**<0.001**
			to		to	2017-2018	0.643	(0.518, 0.799)	<0.001	**<0.001**
			08/03/2020		05/04/2020	2018-2019	0.748	(0.598, 0.935)	0.011	**0.032**
GP	numbers	week 1-10	30/12/2019	week 30-33	20/07/2020	2016-2017	0.944	(0.789, 1.130)	0.529	>0.999
			to		to	2017-2018	0.938	(0.783, 1.123)	0.484	>0.999
			08/03/2020		16/08/2020	2018-2019	0.808	(0.677, 0.964)	0.018	0.054
GP	numbers	week 1-10	30/12/2019	week 50-53	07/12/2020	2016-2017	0.731	(0.599, 0.893)	0.002	**0.006**
			to		to	2017-2018	0.612	(0.504, 0.743)	<0.001	**<0.001**
			08/03/2020		03/01/2021	2018-2019	0.698	(0.571, 0.852)	<0.001	**0.001**
GP	proportion	week 1-10	30/12/2019	week 12-26	16/03/2020	2016-2017	0.731	(0.648, 0.823)	<0.001	**<0.001**
			to		to	2017-2018	0.742	(0.658, 0.836)	<0.001	**<0.001**

			08/03/2020		28/06/2020	2018-2019	0.752 (0.666 , 0.848)	<0.001	**<0.001**
GP	proportion	week 1-10	30/12/2019	week 30-33	20/07/2020	2016-2017	0.944 (0.789 , 1.130)	0.529	>0.999
			to		to	2017-2018	0.938 (0.783 , 1.123)	0.484	>0.999
			08/03/2020		16/08/2020	2018-2019	0.808 (0.677 , 0.964)	0.018	0.054
GP	proportion	week 1-10	30/12/2019	week 50-53	07/12/2020	2016-2017	0.684 (0.566 , 0.825)	<0.001	**<0.001**
			to		to	2017-2018	0.656 (0.544 , 0.791)	<0.001	**<0.001**
			08/03/2020		03/01/2021	2018-2019	0.695 (0.574 , 0.842)	<0.001	**<0.001**
ED	numbers	week 1-10	30/12/2019	week 12-14	16/03/2020	2016-2017	0.528 (0.458 , 0.608)	<0.001	**<0.001**
			to		to	2017-2018	0.568 (0.493 , 0.656)	<0.001	**<0.001**
			08/03/2020		05/04/2020	2018-2019	0.576 (0.498 , 0.666)	<0.001	**<0.001**
ED	proportion	week 1-10	30/12/2019	week 16-17	13/04/2020	2016-2017	1.270 (1.070 , 1.508)	0.006	**0.019**
			to		to	2017-2018	1.426 (1.198 , 1.698)	<0.001	**<0.001**
			08/03/2020		26/04/2020	2018-2019	1.195 (1.006 , 1.420)	0.043	0.129
ED	numbers	week 1-10	30/12/2019	week 30-33	20/07/2020	2016-2017	1.104 (0.988 , 1.234)	0.081	0.244
			to		to	2017-2018	0.978 (0.877 , 1.091)	0.692	>0.999
			08/03/2020		16/08/2020	2018-2019	0.891 (0.799 , 0.994)	0.038	0.115
ED	proportion	week 1-10	30/12/2019	week 41-45	05/10/2020	2016-2017	1.305 (1.161 , 1.467)	<0.001	**<0.001**
			to		to	2017-2018	1.261 (1.123 , 1.415)	<0.001	**<0.001**
			08/03/2020		08/11/2020	2018-2019	1.064 (0.947 , 1.194)	0.295	0.886
ED	numbers	week 1-10	30/12/2019	week 50-53	07/12/2020	2016-2017	0.703 (0.620 , 0.797)	<0.001	**<0.001**
			to		to	2017-2018	0.664 (0.586 , 0.752)	<0.001	**<0.001**
			08/03/2020		03/01/2021	2018-2019	0.635 (0.560 , 0.720)	<0.001	**<0.001**
ED	proportion	week 1-10	30/12/2019	week 53-58	28/12/2020	2016-2017	1.092 (0.981 , 1.216)	0.107	0.321
			to		to	2017-2018	1.158 (1.039 , 1.290)	0.008	**0.024**
			08/03/2020		07/02/2021	2018-2019	0.932 (0.837 , 1.037)	0.196	0.587
ED to HA	numbers	week 1-10	30/12/2019	week 12-14	16/03/2020	2016-2017	0.515 (0.390 , 0.680)	<0.001	**<0.001**
			to		to	2017-2018	0.580 (0.439 , 0.767)	<0.001	**<0.001**
			08/03/2020		05/04/2020	2018-2019	0.669 (0.502 , 0.892)	0.006	**0.018**
ED to HA	numbers	week 1-10	30/12/2019	week 30-33	20/07/2020	2016-2017	1.091 (0.875 , 1.360)	0.437	>0.999
			to		to	2017-2018	0.986 (0.797 , 1.221)	0.901	>0.999
			08/03/2020		16/08/2020	2018-2019	0.965 (0.779 , 1.196)	0.746	>0.999
ED to HA	numbers	week 1-10	30/12/2019	week 50-53	07/12/2020	2016-2017	0.527 (0.402 , 0.691)	<0.001	**<0.001**
			to		to	2017-2018	0.503 (0.385 , 0.656)	<0.001	**<0.001**
			08/03/2020		03/01/2021	2018-2019	0.484 (0.371 , 0.632)	<0.001	**<0.001**
HA	numbers	week 1-10	30/12/2019	week 12-14	16/03/2020	2016-2017	0.561 (0.459 , 0.685)	<0.001	**<0.001**
			to		to	2017-2018	0.583 (0.477 , 0.712)	<0.001	**<0.001**
			08/03/2020		05/04/2020	2018-2019	0.630 (0.510 , 0.779)	<0.001	**<0.001**
HA	proportion	week 1-10	30/12/2019	week 15-33	06/04/2020	2016-2017	1.292 (1.162 , 1.437)	<0.001	**<0.001**

			to		to	2017-2018	1.297 (1.166 , 1.442)	<0.001	**<0.001**
			08/03/2020		16/08/2020	2018-2019	1.128 (1.010 , 1.259)	0.032	0.096
HA	numbers	week 1-10	30/12/2019	week 30-33	20/07/2020	2016-2017	0.931 (0.789 , 1.099)	0.398	>0.999
			to		to	2017-2018	0.945 (0.802 , 1.115)	0.504	>0.999
			08/03/2020		16/08/2020	2018-2019	0.753 (0.638 , 0.889)	<0.001	**0.003**
HA	proportion	week 1-10	30/12/2019	week 41-44	05/10/2020	2016-2017	1.201 (1.018 , 1.418)	0.030	0.091
			to		to	2017-2018	1.256 (1.063 , 1.483)	0.007	**0.022**
			08/03/2020		01/11/2020	2018-2019	0.999 (0.846 , 1.181)	0.993	>0.999
HA	numbers	week 1-10	30/12/2019	week 30-33	20/07/2020	2016-2017	0.522 (0.426 , 0.640)	<0.001	**<0.001**
			to		to	2017-2018	0.453 (0.371 , 0.553)	<0.001	**<0.001**
			08/03/2020		16/08/2020	2018-2019	0.370 (0.303 , 0.453)	<0.001	**<0.001**
Any	numbers	week 36-44	31/08/2020	week 50-53	07/12/2020	2016-2017	0.803 (0.717 , 0.899)	<0.001	**<0.001**
			to		to	2017-2018	0.727 (0.651 , 0.813)	<0.001	**<0.001**
			01/11/2020		03/01/2021	2018-2019	0.768 (0.687 , 0.859)	<0.001	**<0.001**
GP	numbers	week 36-44	31/08/2020	week 50-53	07/12/2020	2016-2017	0.777 (0.634 , 0.952)	0.015	**0.045**
			to		to	2017-2018	0.643 (0.528 , 0.784)	<0.001	**<0.001**
			01/11/2020		03/01/2021	2018-2019	0.732 (0.597 , 0.897)	0.003	**0.008**
ED	numbers	week 36-44	31/08/2020	week 50-53	07/12/2020	2016-2017	0.711 (0.625 , 0.808)	<0.001	**<0.001**
			to		to	2017-2018	0.731 (0.644 , 0.830)	<0.001	**<0.001**
			01/11/2020		03/01/2021	2018-2019	0.756 (0.666 , 0.859)	<0.001	**<0.001**
ED to HA	numbers	week 36-44	31/08/2020	week 50-53	07/12/2020	2016-2017	0.598 (0.453 , 0.788)	<0.001	**<0.001**
			to		to	2017-2018	0.617 (0.471 , 0.809)	<0.001	**0.001**
			01/11/2020		03/01/2021	2018-2019	0.613 (0.468 , 0.803)	<0.001	**0.001**
HA	numbers	week 36-44	31/08/2020	week 50-53	07/12/2020	2016-2017	0.688 (0.559 , 0.848)	<0.001	**0.001**
			to		to	2017-2018	0.570 (0.465 , 0.699)	<0.001	**<0.001**
			01/11/2020		03/01/2021	2018-2019	0.586 (0.478 , 0.717)	<0.001	**<0.001**

* Bonferroni corrected

[a] Period > 1 week represented by the mean of the model coefficients within the period

[b] RRR-ratio of rate ratios for prevalence/incidence outcomes; ROR-ratio of odds ratio for proportion outcomes
