# Supplementary material for: Healthcare contacts with self-harm during COVID-19: An e-cohort whole-population-based study using individual-level linked routine electronic health records in Wales, UK, 2016—March 2021
Source: PLoS One. 2022 Apr 27;17(4):e0266967. doi: 10.1371/journal.pone.0266967 (PMC9045644; doi:10.1371/journal.pone.0266967)
Supplement: S2 Table — Results of linear trends of weekly number and proportion of self-harm contacts and weekly proportion of people with self-harm contacts for primary care (GP), emergency departments (ED) and hospital admissions (HA). (PDF) [file pone.0266967.s016.pdf]

Healthcare contacts with self-harm during COVID-19: an e-cohort whole-population-based study using individual-level linked routine electronic health records in Wales, UK, 2016 – March 2021

Marcos DelPozo-Banos, Sze Chim Lee, Yasmin Friedmann, Ashley Akbari, Fatemeh Torabi, Keith Lloyd, Ronan A Lyons, Ann John

S2 Table. Linear trends for number and proportion of self-harm contacts. Results of linear trends of weekly number and proportion of self-harm contacts and weekly proportion of people with self-harm contacts for primary care (GP), emergency departments (ED) and hospital admissions (HA).

Setting	Outcome (units)	Fitted period	**Slope per month[a] (95% CI)**	p-value of slope	Adjusted p-value **of slope[b]**	p-value for **slope comparisons[c]**
ED	Proportion of contacts (%)	6/2020-3/2021	-1.899 (-2.264, -1.530)	<0.001	**<0.001**	reference
		6/2016-3/2017	-0.158 (-0.479, 0.162)	0.328	>0.999	**0.001**
		6/2017-3/2018	0.109 (-0.163 ,0.380)	0.427	>0.999	**<0.001**
		6/2018-3/2019	0.159 (-0.121 ,0.440)	0.261	>0.999	**<0.001**
		6/2019-3/2020	0.148 (-0.176 ,0.471)	0.366	>0.999	**<0.001**
HA	Number of contacts	6/2020-3/2021	-4.702 (-6.025, -3.380)	<0.001	**<0.001**	reference
		6/2016-3/2017	-2.066 (-3.466, -0.660)	0.005	**0.025**	**0.010**
		6/2017-3/2018	-4.07(-5.655, -2.480)	<0.001	**<0.001**	0.500
		6/2018-3/2019	-0.429 (-1.762 ,0.903)	0.518	>0.999	**<0.001**
		6/2019-3/2020	1.292 (0.64 ,3.220)	0.184	0.919	**<0.001**
HA	Proportions of people (%)	8/2020-3/2021	-0.992 (-1.686, -0.297)	<0.007	**0.034**	reference
		8/2016-3/2017	-0.393 (-0.837, 0.052)	0.081	0.406	0.112
		8/2017-3/2018	-0.649 (-1.147, -0.152)	0.012	0.062	0.362
		8/2018-3/2019	0.385 (-0.083, 0.853)	0.103	0.514	**<0.001**
		8/2019-3/2020	0.192 (-0.390, 0.773)	0.505	>0.999	**0.002**
ED and HA only	Proportions of people (%)	6/2020-12/2020	-1.163 (-1.677, -0.649)	<0.001	**<0.001**	reference
		6/2016-12/2016	-0.046 (-0.420, 0.329)	0.804	>0.999	**<0.001**
		6/2017-12/2017	-0.421 (-0.779, -0.063)	0.023	0.115	**0.008**
		6/2018-12/2018	0.171 (-0.242, 0.583)	0.402	>0.999	**<0.001**
		6/2019-12/2019	0.207 (-0.106 ,0.520)	0.186	0.928	**<0.001**
**ED, HA and GP**	Proportions of people (%)	4/2020-end 12/2020	-0.382 (-0.524, -0.240)	<0.001	**<0.001**	reference
		4/2016-end 12/2016	-0.125 (-0.266, 0.016)	0.081	0.404	**0.006**
		4/2017-end 12/2017	0.049 (-0.089, 0.186)	0.476	>0.999	**<0.001**
		4/2018-end 12/2018	-0.049 (-0.166, 0.068)	0.402	>0.999	**<0.001**

		4/2019-end 12/2019	0.025 (-0.109, 0.159)	0.705	>0.999	**<0.001**
**ED only**	Proportions of people (%)	4/2020-end 2/2021	0.977 (0.639, 1.315)	<0.001	**<0.001**	reference
		4/2016- end 2/2017	0.073 (-0.217, 0.362)	0.616	>0.999	**<0.001**
		4/2017-end 2/2018	0.028 (-0.239, 0.295)	0.834	>0.999	**<0.001**
		4/2018-end 2/2019	-0.362 (-0.617, -0.107)	0.006	0.032	**<0.001**
		4/2019-end 2/2020	-0.610 (-0.885, -0.335)	<0.001	**0.002**	**<0.001**
**HA**	Males aged >24 years, number of contacts	6/2020-12/2020	-3.441 (-4.192, -2.691)	<0.001	**<0.001**	reference
		6/2016-12/2016	-0.405 (1.641 ,0.830)	0.507	>0.999	**<0.001**
		6/2017-12/2017	-0.652 (-1.834, 0.529)	0.267	>0.999	**<0.001**
		6/2018-12/2018	-0.726 (-2.103 ,0.651)	0.289	>0.999	**<0.001**
		6/2019-12/2019	-0.076 (-1.008 ,0.856)	0.868	>0.999	**<0.001**
HA	Females aged >24 years, number of contacts	6/2020-12/2020	-2.056 (-3.331, -0.782)	0.003	0.013	reference
		6/2016-12/2016	-2.992 (-4.296, -1.687)	<0.001	<0.001	0.246
		6/2017-12/2017	-1.879 (-3.117, -0.641)	0.004	0.022	0.825
		6/2018-12/2018	0.038 (-0.913, 0.989)	0.935	>0.999	0.01
		6/2019-12/2019	-1.408 (-2.411, -0.405)	0.008	0.038	0.420

[a] Slope and CI columns holds the monthly change in the weekly outcomes and the confidence interval

[b] Bonferroni adjusted

[c] p-value for the interaction terms between time and counterfactual period. P<0.05 for a counterfactual period means that the slope for that year is different than that in our COVID-19 reference period.
