# Supplementary material for: Healthcare contacts with self-harm during COVID-19: An e-cohort whole-population-based study using individual-level linked routine electronic health records in Wales, UK, 2016—March 2021
Source: PLoS One. 2022 Apr 27;17(4):e0266967. doi: 10.1371/journal.pone.0266967 (PMC9045644; doi:10.1371/journal.pone.0266967)
Supplement: S3 Table — Summary of RORs comparing change in proportion of people who self-harmed and were in contact with primary care (GP), emergency departments (ED) and/or hospital admissions (HA) between reference and target periods to the respective changes in previous years. (PDF) [file pone.0266967.s017.pdf]

Healthcare contacts with self-harm during COVID-19: an e-cohort whole-population-based study using individual-level linked routine electronic health records in Wales, UK, 2016 – March 2021

Marcos DelPozo-Banos, Sze Chim Lee, Yasmin Friedmann, Ashley Akbari, Fatemeh Torabi, Keith Lloyd, Ronan A Lyons, Ann John

S3 Table. RORs of people in contact with one or more healthcare settings with self-harm. Summary of RORs comparing change in proportion of people who self-harmed and were in contact with primary care (GP), emergency departments (ED) and/or hospital admissions (HA) between reference and target periods to the respective changes in previous years.

Setting	Reference period[a]		Target period[a]		Year as counterfactual	RRR/ROR[b]	95% CI	*p*-value	*p*-value*
GP	week 1-10	30/12/2019	week 14-18	30/03/2020	2016-2017	1.198	(1.048 , 1.370)	0.008	**0.025**
		to		to	2017-2018	1.211	(1.056 , 1.388)	0.006	**0.018**
		08/03/2020		03/05/2020	2018-2019	1.301	(1.135 , 1.493)	<0.001	**<0.001**
ED	week 1-10	30/12/2019	week 28-33	06/07/2020	2016-2017	1.165	(1.085 , 1.251)	<0.001	**<0.001**
		to		to	2017-2018	1.096	(1.023 , 1.175)	0.009	**0.027**
		08/03/2020		16/08/2020	2018-2019	1.117	(1.041 , 1.198)	0.002	**0.006**
HA	week 1-10	30/12/2019	week 58-61	01/02/2021	2016-2017	0.705	(0.605 , 0.821)	<0.001	**<0.001**
		to		to	2017-2018	0.830	(0.707 , 0.975)	0.023	0.070
		08/03/2020		28/02/2021	2018-2019	0.591	(0.508 , 0.688)	<0.001	**<0.001**
GP only	week 1-10	30/12/2019	week 14-18	30/03/2020	2016-2017	1.151	(0.957 , 1.383)	0.135	0.405
		to		to	2017-2018	1.198	(0.992 , 1.446)	0.061	0.183
		08/03/2020		03/05/2020	2018-2019	1.291	(1.073 , 1.553)	0.007	**0.020**
GP only	week 1-10	30/12/2019	week 28-33	06/07/2020	2016-2017	0.790	(0.659 , 0.947)	0.011	**0.032**
		to		to	2017-2018	0.791	(0.660 , 0.948)	0.011	**0.033**
		08/03/2020		16/08/2020	2018-2019	0.875	(0.733 , 1.044)	0.139	0.416
ED only	week 1-10	30/12/2019	week 12-15	16/03/2020	2016-2017	0.905	(0.786 , 1.041)	0.162	0.487
		to		to	2017-2018	0.916	(0.794 , 1.056)	0.225	0.674
		08/03/2020		12/04/2021	2018-2019	0.830	(0.720 , 0.957)	0.010	**0.031**
ED only	week 1-10	30/12/2019	week 50-53	07/12/2020	2016-2017	1.243	(1.105 , 1.400)	<0.001	**<0.001**
		to		to	2017-2018	1.297	(1.150 , 1.463)	<0.001	**<0.001**
		08/03/2020		03/01/2021	2018-2019	1.283	(1.136 , 1.449)	<0.001	**<0.001**
ED only	week 1-10	30/12/2019	week 58-61	01/02/2021	2016-2017	1.254	(1.120 , 1.404)	<0.001	**<0.001**
		to		to	2017-2018	1.228	(1.092 , 1.380)	<0.001	**0.002**
		08/03/2020		28/02/2021	2018-2019	1.328	(1.180 , 1.495)	<0.001	**<0.001**
GP, ED & HA only	week 1-10	30/12/2019	week 14-18	30/03/2020	2016-2017	2.101	(1.280 , 3.447)	0.003	**0.010**
		to		to	2017-2018	2.794	(1.589 , 4.912)	<0.001	**0.001**
		08/03/2020		03/05/2020	2018-2019	2.038	(1.231 , 3.372)	0.006	**0.017**
GP, ED & HA only	week 1-10	30/12/2019	week 28-33	06/07/2020	2016-2017	1.939	(1.215 , 3.094)	0.005	**0.016**
		to		to	2017-2018	1.865	(1.161 , 2.996)	0.010	**0.030**

	08/03/2020	16/08/2020	2018-2019	2.550	(	1.577	,	4.121	)	<0.001	**<0.001**

* Bonferroni corrected

[a] Period > 1 week represented by the mean of the model coefficients within the period

[b] RRR-ratio of rate ratios for prevalence/incidence outcomes; ROR-ratio of odds ratio for proportion outcomes
