# Supplementary material for: Healthcare contacts with self-harm during COVID-19: An e-cohort whole-population-based study using individual-level linked routine electronic health records in Wales, UK, 2016—March 2021
Source: PLoS One. 2022 Apr 27;17(4):e0266967. doi: 10.1371/journal.pone.0266967 (PMC9045644; doi:10.1371/journal.pone.0266967)
Supplement: S4 Table — Summary of RRR/RORs based on the difference-in-difference (DiD) approach comparing changes in number and proportion of self-harm contacts per method across primary care (GP), emergency departments (ED) and hospital admissions (HA) between reference and target periods to the respective changes in previous years (as counterfactual). (PDF) [file pone.0266967.s018.pdf]

Healthcare contacts with self-harm during COVID-19: an e-cohort whole-population-based study using individual-level linked routine electronic health records in Wales, UK, 2016 – March 2021

Marcos DelPozo-Banos, Sze Chim Lee, Yasmin Friedmann, Ashley Akbari, Fatemeh Torabi, Keith Lloyd, Ronan A Lyons, Ann John

S4 Table. RORs/RRRs of healthcare service contacts with self-harm in each setting stratified by method. Summary of RRR/RORs based on the difference-in-difference (DiD) approach comparing changes in number and proportion of self-harm contacts per method across primary care (GP), emergency departments (ED) and hospital admissions (HA) between reference and target periods to the respective changes in previous years (as counterfactual).

				Wave 1				Wave 2			
				Reference period: week 1-10 (30/12/2019-08/03/2020)				Reference period: week 1-10 (30/12/2019-08/03/2020)			
	Method of		Year as	Target period: week 11-33 (03/09/2019-16/08/2020)				Target period: week 34 onwards (17/08/2019 onwards)			
Setting	Self-harm	Outcome	counterfactual	RRR/ROR	95% CI	*p*-value	*p*-value*	RRR/ROR	95% CI	*p*-value	*p*-value*
ED	Burning	numbers	2016	2.667	(0.492 , 14.461)	0.256	>0.999	2.000	(0.271 , 14.784)	0.497	>0.999
			2017	1.167	(0.208 , 6.559)	0.861	>0.999	0.833	(0.114 , 6.111)	0.858	>0.999
			2018	1.037	(0.173 , 6.233)	0.968	>0.999	2.000	(0.194 , 20.614)	0.560	>0.999
			2019	2.800	(0.463 , 16.929)	0.262	>0.999	2.000	(0.241 , 16.612)	0.521	>0.999
ED		proportion	2016	3.122	(0.576 , 16.912)	0.187	0.746	1.952	(0.264 , 14.416)	0.512	>0.999
			2017	1.495	(0.266 , 8.398)	0.648	>0.999	0.862	(0.118 , 6.314)	0.884	>0.999
			2018	1.272	(0.212 , 7.639)	0.792	>0.999	2.049	(0.199 , 21.103)	0.546	>0.999
			2019	3.291	(0.545 , 19.876)	0.194	0.777	1.827	(0.220 , 15.159)	0.577	>0.999
ED	Injuries	numbers	2016	1.054	(0.845 , 1.316)	0.640	>0.999	1.016	(0.804 , 1.283)	0.896	>0.999
			2017	0.909	(0.728 , 1.134)	0.396	>0.999	1.052	(0.831 , 1.333)	0.672	>0.999
			2018	0.736	(0.586 , 0.924)	0.008	**0.033**	0.815	(0.640 , 1.038)	0.098	0.391
			2019	0.662	(0.523 , 0.838)	<0.001	**0.002**	0.983	(0.760 , 1.271)	0.896	>0.999
ED		proportion	2016	1.234	(0.999 , 1.526)	0.052	0.206	0.992	(0.792 , 1.241)	0.941	>0.999
			2017	1.165	(0.942 , 1.440)	0.159	0.636	1.089	(0.867 , 1.366)	0.464	>0.999
			2018	0.903	(0.726 , 1.124)	0.362	>0.999	0.835	(0.662 , 1.054)	0.129	0.518
			2019	0.778	(0.620 , 0.977)	0.030	0.122	0.898	(0.700 , 1.151)	0.396	>0.999
ED	Poisoning	numbers	2016	0.826	(0.703 , 0.972)	0.021	0.084	0.887	(0.749 , 1.051)	0.167	0.667
			2017	0.761	(0.647 , 0.895)	<0.001	**0.004**	0.966	(0.812 , 1.148)	0.691	>0.999
			2018	0.829	(0.703 , 0.978)	0.027	0.106	0.898	(0.755 , 1.068)	0.222	0.889
			2019	0.860	(0.732 , 1.012)	0.069	0.276	1.043	(0.878 , 1.239)	0.633	>0.999
ED		proportion	2016	0.968	(0.834 , 1.123)	0.664	>0.999	0.866	(0.741 , 1.012)	0.071	0.283
			2017	0.975	(0.841 , 1.132)	0.744	>0.999	0.999	(0.852 , 1.171)	0.989	>0.999
			2018	1.018	(0.873 , 1.185)	0.823	>0.999	0.920	(0.783 , 1.080)	0.307	>0.999
			2019	1.011	(0.871 , 1.174)	0.882	>0.999	0.953	(0.812 , 1.117)	0.551	>0.999
GP	Hanging	numbers	2016	2.667	(0.892 , 7.976)	0.079	0.317	1.091	(0.304 , 3.910)	0.894	>0.999
			2017	1.123	(0.377 , 3.341)	0.835	>0.999	0.571	(0.157 , 2.074)	0.395	>0.999
			2018	1.504	(0.479 , 4.728)	0.485	>0.999	0.667	(0.177 , 2.517)	0.550	>0.999
			2019	1.778	(0.567 , 5.577)	0.324	>0.999	1.143	(0.284 , 4.595)	0.851	>0.999
GP		proportion	2016	3.061	(1.027 , 9.127)	0.045	0.179	1.086	(0.304 , 3.880)	0.899	>0.999

			2017	1.323	(0.446 , 3.925)	0.614	>0.999	0.562	(0.155 , 2.033)	0.380	>0.999
			2018	1.699	(0.542 , 5.323)	0.363	>0.999	0.649	(0.172 , 2.444)	0.523	>0.999
			2019	2.063	(0.660 , 6.451)	0.213	0.853	1.098	(0.274 , 4.402)	0.895	>0.999
GP	Injuries	numbers	2016	0.908	(0.763 , 1.081)	0.276	>0.999	1.076	(0.901 , 1.285)	0.418	>0.999
			2017	0.864	(0.726 , 1.029)	0.101	0.406	1.029	(0.861 , 1.229)	0.756	>0.999
			2018	0.855	(0.718 , 1.018)	0.078	0.313	1.071	(0.896 , 1.281)	0.449	>0.999
			2019	0.783	(0.659 , 0.931)	0.006	**0.022**	1.093	(0.914 , 1.307)	0.330	>0.999
GP		proportion	2016	1.042	(0.895 , 1.213)	0.597	>0.999	1.071	(0.918 , 1.250)	0.384	>0.999
			2017	1.019	(0.874 , 1.186)	0.813	>0.999	1.011	(0.867 , 1.180)	0.886	>0.999
			2018	0.966	(0.830 , 1.124)	0.652	>0.999	1.043	(0.894 , 1.218)	0.591	>0.999
			2019	0.909	(0.782 , 1.056)	0.213	0.853	1.050	(0.899 , 1.226)	0.538	>0.999
GP	Poisoning	numbers	2016	0.865	(0.781 , 0.958)	0.005	**0.022**	0.991	(0.891 , 1.102)	0.863	>0.999
			2017	0.849	(0.767 , 0.941)	0.002	**0.007**	1.054	(0.947 , 1.173)	0.340	>0.999
			2018	0.897	(0.808 , 0.996)	0.041	0.164	1.018	(0.914 , 1.135)	0.742	>0.999
			2019	0.887	(0.800 , 0.984)	0.023	0.094	1.030	(0.925 , 1.147)	0.591	>0.999
GP		proportion	2016	0.993	(0.938 , 1.051)	0.811	>0.999	0.986	(0.929 , 1.047)	0.645	>0.999
			2017	1.001	(0.945 , 1.060)	0.979	>0.999	1.036	(0.975 , 1.101)	0.256	>0.999
			2018	1.013	(0.954 , 1.075)	0.675	>0.999	0.992	(0.932 , 1.055)	0.794	>0.999
			2019	1.030	(0.971 , 1.092)	0.327	>0.999	0.990	(0.931 , 1.052)	0.736	>0.999
HA	Burning	numbers	2016	0.857	(0.189 , 3.888)	0.842	>0.999	0.107	(0.010 , 1.121)	0.062	0.249
			2017	0.333	(0.062 , 1.779)	0.199	0.794	0.086	(0.007 , 1.084)	0.058	0.231
			2018	0.700	(0.168 , 2.910)	0.624	>0.999	0.111	(0.011 , 1.127)	0.063	0.252
			2019	0.571	(0.147 , 2.228)	0.420	>0.999	0.190	(0.018 , 1.992)	0.166	0.665
HA		proportion	2016	0.987	(0.218 , 4.470)	0.987	>0.999	0.121	(0.012 , 1.267)	0.078	0.312
			2017	0.394	(0.074 , 2.100)	0.275	>0.999	0.096	(0.008 , 1.207)	0.070	0.278
			2018	0.786	(0.189 , 3.259)	0.740	>0.999	0.131	(0.013 , 1.331)	0.086	0.343
			2019	0.525	(0.135 , 2.043)	0.353	>0.999	0.176	(0.017 , 1.835)	0.146	0.585
HA	Hanging	numbers	2016	1.086	(0.537 , 2.195)	0.819	>0.999	0.584	(0.289 , 1.178)	0.133	0.532
			2017	1.052	(0.536 , 2.064)	0.883	>0.999	0.992	(0.481 , 2.047)	0.983	>0.999
			2018	1.503	(0.760 , 2.974)	0.241	0.966	0.960	(0.480 , 1.922)	0.908	>0.999
			2019	1.425	(0.744 , 2.729)	0.285	>0.999	1.159	(0.583 , 2.301)	0.674	>0.999
HA		proportion	2016	1.251	(0.622 , 2.517)	0.530	>0.999	0.661	(0.329 , 1.327)	0.244	0.976
			2017	1.244	(0.637 , 2.429)	0.523	>0.999	1.106	(0.539 , 2.269)	0.784	>0.999
			2018	1.687	(0.857 , 3.322)	0.130	0.520	1.136	(0.570 , 2.261)	0.717	>0.999
			2019	1.310	(0.687 , 2.496)	0.412	>0.999	1.069	(0.541 , 2.112)	0.848	>0.999
HA	Injuries	numbers	2016	0.828	(0.651 , 1.053)	0.125	0.499	0.841	(0.656 , 1.079)	0.174	0.694
			2017	0.734	(0.580 , 0.928)	0.010	**0.039**	0.953	(0.743 , 1.222)	0.703	>0.999
			2018	0.785	(0.623 , 0.989)	0.040	0.159	0.924	(0.725 , 1.177)	0.521	>0.999
			2019	0.902	(0.717 , 1.133)	0.375	>0.999	1.053	(0.828 , 1.339)	0.674	>0.999
HA		proportion	2016	0.954	(0.761 , 1.197)	0.685	>0.999	0.953	(0.755 , 1.202)	0.683	>0.999
			2017	0.868	(0.696 , 1.081)	0.206	0.825	1.062	(0.841 , 1.341)	0.614	>0.999

			2018	0.881	(	0.709	,	1.094)	0.250	>0.999	1.093	(	0.871	,	1.371)	0.445	>0.999
			2019	0.829	(	0.669	,	1.026)	0.085	0.341	0.971	(	0.776	,	1.216)	0.799	>0.999
HA	Poisoning	numbers	2016	0.895	(	0.818	,	0.979)	0.016	0.063	0.912	(	0.829	,	1.005)	0.062	0.248
			2017	0.852	(	0.779	,	0.932)	<0.001	**0.002**	0.898	(	0.815	,	0.989)	0.029	0.116
			2018	0.918	(	0.839	,	1.005)	0.064	0.255	0.847	(	0.770	,	0.932)	<0.001	**0.003**
			2019	1.121	(	1.025	,	1.226)	0.013	0.050	1.108	(	1.007	,	1.220)	0.035	0.142
HA		proportion	2016	1.031	(	0.993	,	1.072)	0.113	0.452	1.033	(	0.991	,	1.077)	0.126	0.504
			2017	1.007	(	0.969	,	1.047)	0.718	>0.999	1.001	(	0.960	,	1.044)	0.965	>0.999
			2018	1.030	(	0.990	,	1.073)	0.142	0.566	1.002	(	0.960	,	1.045)	0.928	>0.999
			2019	1.030	(	0.990	,	1.072)	0.140	0.561	1.022	(	0.980	,	1.067)	0.308	>0.999
HA	Jumping	numbers	2016	0.208	(	0.075	,	0.574)	0.002	**0.010**	0.277	(	0.098	,	0.782)	0.015	0.062
			2017	0.428	(	0.155	,	1.177)	0.100	0.401	0.428	(	0.155	,	1.177)	0.100	0.401
			2018	0.684	(	0.268	,	1.750)	0.429	>0.999	0.684	(	0.268	,	1.750)	0.429	>0.999
			2019	0.640	(	0.270	,	1.515)	0.310	>0.999	1.067	(	0.425	,	2.675)	0.891	>0.999
HA		proportion	2016	0.239	(	0.087	,	0.659)	0.006	**0.023**	0.314	(	0.111	,	0.883)	0.028	0.112
			2017	0.506	(	0.184	,	1.388)	0.186	0.742	0.477	(	0.174	,	1.307)	0.150	0.601
			2018	0.768	(	0.302	,	1.957)	0.581	>0.999	0.810	(	0.318	,	2.062)	0.658	>0.999
			2019	0.588	(	0.249	,	1.388)	0.226	0.902	0.984	(	0.394	,	2.458)	0.973	>0.999

* Bonferroni corrected
