# Supplementary material for: Healthcare contacts with self-harm during COVID-19: An e-cohort whole-population-based study using individual-level linked routine electronic health records in Wales, UK, 2016—March 2021
Source: PLoS One. 2022 Apr 27;17(4):e0266967. doi: 10.1371/journal.pone.0266967 (PMC9045644; doi:10.1371/journal.pone.0266967)
Supplement: S5 Table — Summary of RORs and RRRs for self-harm contacts stratified by age and sex for primary care (GP), emergency departments (ED) and hospital admissions (HA). (PDF) [file pone.0266967.s019.pdf]

Healthcare contacts with self-harm during COVID-19: an e-cohort whole-population-based study using individual-level linked routine electronic health records in Wales, UK, 2016 – March 2021

Marcos DelPozo-Banos, Sze Chim Lee, Yasmin Friedmann, Ashley Akbari, Fatemeh Torabi, Keith Lloyd, Ronan A Lyons, Ann John

S5 Table. RORs/RRRs of healthcare service contacts with self-harm in any and each setting stratified by sex and age. Summary of RORs and RRRs for self-harm contacts stratified by age and sex for primary care (GP), emergency departments (ED) and hospital admissions (HA).

Setting	Outcome	Variable	Category	Reference period (pre-COVID)[a]		Target period (post-COVID)[a]		Year as counterfactual	RRR/ROR[b]	95% CI	*p*-value	*p*-value*
Any	numbers	Age group	(10-24) vs. (>24)**	week 1-10	30/12/2019	week 11-33	09/03/2020	2016-2017	0.859	(0.754, 0.978)	0.022	0.065
		(years)			to		to	2017-2018	0.869	(0.763, 0.990)	0.035	0.104
					08/03/2020		16/08/2020	2018-2019	0.786	(0.689, 0.898)	<0.001	**0.001**
		Sex	Female vs. Male**					2016-2017	0.902	(0.794, 1.025)	0.113	0.340
								2017-2018	1.047	(0.921, 1.189)	0.483	>0.999
								2018-2019	1.009	(0.886, 1.149)	0.894	>0.999
Any	numbers	Age group	(10-24) vs. (>24)	week 1-10	30/12/2019	week 34-63	17/08/2020	2016-2017	1.073	(0.947, 1.216)	0.267	0.800
		(years)			to		to	2017-2018	1.080	(0.954, 1.224)	0.225	0.674
					08/03/2020		14/03/2021	2018-2019	1.037	(0.913, 1.178)	0.574	>0.999
		Sex	Female vs. Male					2016-2017	1.149	(1.016, 1.300)	0.027	0.082
								2017-2018	1.205	(1.065, 1.364)	0.003	**0.009**
								2018-2019	1.188	(1.048, 1.348)	0.007	**0.021**
Any	proportion	Age group	(10-24) vs. (>24)	week 1-10	30/12/2019	week 11-33	09/03/2020	2016-2017	0.948	(0.841, 1.067)	0.375	>0.999
		(years)			to		to	2017-2018	1.034	(0.919, 1.165)	0.576	>0.999
					08/03/2020		16/08/2020	2018-2019	0.928	(0.822, 1.049)	0.232	0.697
		Sex	Female vs. Male					2016-2017	0.866	(0.770, 0.975)	0.017	0.051
								2017-2018	1.004	(0.893, 1.129)	0.947	>0.999
								2018-2019	1.010	(0.895, 1.140)	0.870	>0.999
Any	proportion	Age group	(10-24) vs. (>24)	week 1-10	30/12/2019	week 34-63	17/08/2020	2016-2017	1.097	(0.979, 1.229)	0.111	0.334
		(years)			to		to	2017-2018	1.167	(1.041, 1.308)	0.008	**0.025**
					08/03/2020		14/03/2021	2018-2019	1.147	(1.021, 1.289)	0.021	0.064
		Sex	Female vs. Male					2016-2017	1.108	(0.989, 1.241)	0.077	0.232
								2017-2018	1.151	(1.027, 1.290)	0.016	**0.048**
								2018-2019	1.176	(1.047, 1.321)	0.006	**0.019**
GP	numbers	Age group	(10-24) vs. (>24)	week 1-10	30/12/2019	week 11-33	09/03/2020	2016-2017	0.868	(0.691, 1.090)	0.224	0.672
		(years)			to		to	2017-2018	0.812	(0.647, 1.020)	0.074	0.221
					08/03/2020		16/08/2020	2018-2019	0.807	(0.642, 1.016)	0.068	0.203
		Sex	Female vs. Male					2016-2017	0.803	(0.637, 1.013)	0.064	0.192
								2017-2018	0.933	(0.741, 1.176)	0.557	>0.999
								2018-2019	0.921	(0.729, 1.163)	0.489	>0.999
GP	numbers	Age group	(10-24) vs. (>24)	week 1-10	30/12/2019	week 34-63	17/08/2020	2016-2017	1.038	(0.834, 1.291)	0.738	>0.999
		(years)			to		to	2017-2018	1.041	(0.837, 1.295)	0.715	>0.999
					08/03/2020		14/03/2021	2018-2019	1.059	(0.850, 1.321)	0.608	>0.999
		Sex	Female vs. Male					2016-2017	1.158	(0.926, 1.447)	0.198	0.595

								2017-2018	1.159 (0.927 , 1.449)	0.195	0.584
								2018-2019	1.206 (0.963 , 1.511)	0.103	0.309
GP	proportion	Age group (years)	(10-24) vs. (>24)	week 1-10	30/12/2019 to 08/03/2020	week 11-33	09/03/2020 to 16/08/2020	2016-2017	0.984 (0.788 , 1.229)	0.890	>0.999
								2017-2018	0.960 (0.769 , 1.199)	0.720	>0.999
								2018-2019	0.923 (0.738 , 1.156)	0.487	>0.999
		Sex	Female vs. Male					2016-2017	0.799 (0.637 , 1.002)	0.052	0.155
								2017-2018	0.924 (0.737 , 1.158)	0.493	>0.999
								2018-2019	0.933 (0.742 , 1.173)	0.551	>0.999
GP	proportion	Age group (years)	(10-24) vs. (>24)	week 1-10	30/12/2019 to 08/03/2020	week 34-63	17/08/2020 to 14/03/2021	2016-2017	1.062 (0.858 , 1.313)	0.582	>0.999
								2017-2018	1.121 (0.906 , 1.387)	0.292	0.876
								2018-2019	1.116 (0.900 , 1.384)	0.318	0.955
		Sex	Female vs. Male					2016-2017	1.138 (0.915 , 1.414)	0.245	0.736
								2017-2018	1.121 (0.902 , 1.395)	0.303	0.908
								2018-2019	1.194 (0.958 , 1.488)	0.115	0.345
ED	numbers	Age group (years)	(10-24) vs. (>24)	week 1-10	30/12/2019 to 08/03/2020	week 11-33	09/03/2020 to 16/08/2020	2016-2017	0.840 (0.724 , 0.976)	0.022	0.067
								2017-2018	0.837 (0.720 , 0.971)	0.019	0.058
								2018-2019	0.781 (0.672 , 0.909)	0.001	**0.004**
		Sex	Female vs. Male					2016-2017	0.883 (0.763 , 1.022)	0.094	0.283
								2017-2018	1.064 (0.920 , 1.231)	0.404	>0.999
								2018-2019	0.971 (0.838 , 1.125)	0.698	>0.999
ED	numbers	Age group (years)	(10-24) vs. (>24)	week 1-10	30/12/2019 to 08/03/2020	week 34-63	17/08/2020 to 14/03/2021	2016-2017	1.054 (0.913 , 1.217)	0.475	>0.999
								2017-2018	0.923 (0.799 , 1.066)	0.277	0.830
								2018-2019	0.972 (0.840 , 1.125)	0.703	>0.999
		Sex	Female vs. Male					2016-2017	1.021 (0.886 , 1.176)	0.777	>0.999
								2017-2018	1.132 (0.982 , 1.304)	0.088	0.264
								2018-2019	1.020 (0.884 , 1.178)	0.782	>0.999
ED	proportion	Age group (years)	(10-24) vs. (>24)	week 1-10	30/12/2019 to 08/03/2020	week 11-33	09/03/2020 to 16/08/2020	2016-2017	1.107 (0.939 , 1.306)	0.226	0.678
								2017-2018	1.173 (0.994 , 1.385)	0.060	0.179
								2018-2019	1.120 (0.946 , 1.325)	0.190	0.570
		Sex	Female vs. Male					2016-2017	0.869 (0.739 , 1.022)	0.089	0.267
								2017-2018	1.099 (0.935 , 1.293)	0.251	0.754
								2018-2019	1.017 (0.862 , 1.199)	0.845	>0.999
ED	proportion	Age group (years)	(10-24) vs. (>24)	week 1-10	30/12/2019 to 08/03/2020	week 34-63	17/08/2020 to 14/03/2021	2016-2017	1.234 (1.053 , 1.445)	0.009	**0.028**
								2017-2018	1.071 (0.913 , 1.255)	0.401	>0.999
								2018-2019	1.273 (1.083 , 1.496)	0.003	**0.010**
		Sex	Female vs. Male					2016-2017	0.957 (0.819 , 1.119)	0.584	>0.999
								2017-2018	1.146 (0.980 , 1.341)	0.088	0.264
								2018-2019	1.052 (0.897 , 1.233)	0.534	>0.999
ED to HA	numbers	Age group (years)	(10-24) vs. (>24)	week 1-10	30/12/2019 to 08/03/2020	week 11-33	09/03/2020 to 16/08/2020	2016-2017	0.912 (0.674 , 1.234)	0.550	>0.999
								2017-2018	0.790 (0.585 , 1.068)	0.125	0.376
								2018-2019	0.815 (0.604 , 1.100)	0.181	0.543
		Sex	Female vs. Male					2016-2017	0.850 (0.629 , 1.149)	0.291	0.873
								2017-2018	1.002 (0.746 , 1.347)	0.988	>0.999
								2018-2019	0.897 (0.667 , 1.205)	0.468	>0.999

ED to HA	numbers	Age group (years)	(10-24) vs. (>24)	week 1-10	30/12/2019 to 08/03/2020	week 34-63	17/08/2020 to 14/03/2021	2016-2017	1.276 (0.947 , 1.720)	0.109	0.326
								2017-2018	0.972 (0.723 , 1.307)	0.852	>0.999
								2018-2019	1.116 (0.831 , 1.498)	0.465	>0.999
		Sex	Female vs. Male					2016-2017	1.118 (0.828 , 1.509)	0.467	>0.999
								2017-2018	1.174 (0.874 , 1.577)	0.288	0.863
								2018-2019	0.914 (0.681 , 1.226)	0.548	>0.999
ED to HA	proportion	Age group (years)	(10-24) vs. (>24)	week 1-10	30/12/2019 to 08/03/2020	week 11-33	09/03/2020 to 16/08/2020	2016-2017	1.168 (0.914 , 1.491)	0.215	0.644
								2017-2018	0.943 (0.740 , 1.201)	0.632	>0.999
								2018-2019	1.063 (0.838 , 1.350)	0.615	>0.999
		Sex	Female vs. Male					2016-2017	0.969 (0.757 , 1.241)	0.805	>0.999
								2017-2018	0.966 (0.760 , 1.229)	0.781	>0.999
								2018-2019	0.896 (0.706 , 1.137)	0.367	>0.999
ED to HA	proportion	Age group (years)	(10-24) vs. (>24)	week 1-10	30/12/2019 to 08/03/2020	week 34-63	17/08/2020 to 14/03/2021	2016-2017	1.278 (1.001 , 1.630)	0.049	0.146
								2017-2018	1.061 (0.834 , 1.350)	0.628	>0.999
								2018-2019	1.169 (0.923 , 1.481)	0.195	0.584
		Sex	Female vs. Male					2016-2017	1.104 (0.861 , 1.415)	0.437	>0.999
								2017-2018	1.048 (0.822 , 1.336)	0.704	>0.999
								2018-2019	0.844 (0.664 , 1.072)	0.165	0.495
HA	numbers	Age group (years)	(10-24) vs. (>24)	week 1-10	30/12/2019 to 08/03/2020	week 11-33	09/03/2020 to 16/08/2020	2016-2017	0.886 (0.713 , 1.102)	0.278	0.835
								2017-2018	0.951 (0.766 , 1.181)	0.650	>0.999
								2018-2019	0.813 (0.648 , 1.021)	0.075	0.226
		Sex	Female vs. Male					2016-2017	0.986 (0.791 , 1.228)	0.899	>0.999
								2017-2018	1.023 (0.823 , 1.272)	0.837	>0.999
								2018-2019	1.075 (0.856 , 1.350)	0.535	>0.999
HA	numbers	Age group (years)	(10-24) vs. (>24)	week 1-10	30/12/2019 to 08/03/2020	week 34-63	17/08/2020 to 14/03/2021	2016-2017	1.363 (1.100 , 1.690)	0.005	**0.014**
								2017-2018	1.473 (1.188 , 1.825)	<0.001	**0.001**
								2018-2019	1.315 (1.053 , 1.642)	0.016	**0.047**
		Sex	Female vs. Male					2016-2017	1.510 (1.214 , 1.879)	<0.001	**<0.001**
								2017-2018	1.396 (1.123 , 1.737)	0.003	**0.008**
								2018-2019	1.415 (1.130 , 1.772)	0.003	**0.008**
HA	proportion	Age group (years)	(10-24) vs. (>24)	week 1-10	30/12/2019 to 08/03/2020	week 11-33	09/03/2020 to 16/08/2020	2016-2017	0.867 (0.702 , 1.071)	0.185	0.556
								2017-2018	0.950 (0.770 , 1.172)	0.630	>0.999
								2018-2019	0.827 (0.664 , 1.032)	0.092	0.277
		Sex	Female vs. Male					2016-2017	0.915 (0.739 , 1.135)	0.420	>0.999
								2017-2018	0.941 (0.761 , 1.164)	0.577	>0.999
								2018-2019	0.976 (0.781 , 1.219)	0.828	>0.999
HA	proportion	Age group (years)	(10-24) vs. (>24)	week 1-10	30/12/2019 to 08/03/2020	week 34-63	17/08/2020 to 14/03/2021	2016-2017	1.357 (1.103 , 1.670)	0.004	**0.012**
								2017-2018	1.458 (1.185 , 1.794)	<0.001	**0.001**
								2018-2019	1.382 (1.114 , 1.713)	0.003	**0.010**
		Sex	Female vs. Male					2016-2017	1.502 (1.214 , 1.857)	<0.001	**<0.001**
								2017-2018	1.329 (1.074 , 1.644)	0.009	**0.026**
								2018-2019	1.349 (1.082 , 1.680)	0.008	**0.023**

[a] Period > 1 week represented by the mean of the model coefficients within the period
[b] RRR-ratio of rate ratios for prevalence/incidence outcomes; ROR-ratio of odds ratio for proportion outcomes
* Bonferroni corrected

** Reference group: >24 year, male
