# Supplementary material for: Healthcare contacts with self-harm during COVID-19: An e-cohort whole-population-based study using individual-level linked routine electronic health records in Wales, UK, 2016—March 2021
Source: PLoS One. 2022 Apr 27;17(4):e0266967. doi: 10.1371/journal.pone.0266967 (PMC9045644; doi:10.1371/journal.pone.0266967)
Supplement: S6 Table — Summary of RORs comparing change in proportion of people who self-harm and are in contact with primary care (GP), emergency departments (ED) and/or hospital admissions (HA) between reference and target periods to the respective changes in previous years stratified by age and sex. (PDF) [file pone.0266967.s020.pdf]

Healthcare contacts with self-harm during COVID-19: an e-cohort whole-population-based study using individual-level linked routine electronic health records in Wales, UK, 2016 – March 2021

Marcos DelPozo-Banos, Sze Chim Lee, Yasmin Friedmann, Ashley Akbari, Fatemeh Torabi, Keith Lloyd, Ronan A Lyons, Ann John

S6 Table. RORs of people in contact with one or more healthcare settings with self-harm stratified by sex and age. Summary of RORs comparing change in proportion of people who self-harm and are in contact with primary care (GP), emergency departments (ED) and/or hospital admissions (HA) between reference and target periods to the respective changes in previous years stratified by age and sex.

Setting	Variable	Category	Reference period[a]		Target period[a]		Year as counterfactual	RRR/ROR[b]	95% CI					*p*-value	*p*-value*
ED only	Age group (years)	(10-24) vs. (>24)	week 1-10	30/12/2019 to 08/03/2020	week 50-53	07/12/2020 to 03/01/2021	2016-2017	0.760	(	0.585	,	0.986	)	0.039	0.117
							2017-2018	0.705	(	0.542	,	0.916	)	0.009	**0.027**
							2018-2019	0.743	(	0.568	,	0.972	)	0.030	0.091
	Sex	Female vs. Male					2016-2017	0.741	(	0.585	,	0.939	)	0.013	**0.039**
							2017-2018	0.927	(	0.730	,	1.179	)	0.537	>0.999
							2018-2019	0.886	(	0.695	,	1.129	)	0.328	0.984
HA only	Age group (years)	(10-24) vs. (>24)	week 1-10	30/12/2019 to 08/03/2020	week 14-18	30/03/2020 to 03/05/2020	2016-2017	1.437	(	1.040	,	1.987	)	0.028	0.084
							2017-2018	1.681	(	1.212	,	2.333	)	0.002	**0.006**
							2018-2019	1.350	(	0.968	,	1.883	)	0.077	0.232
	Sex	Female vs. Male					2016-2017	1.692	(	1.227	,	2.333	)	0.001	**0.004**
							2017-2018	1.355	(	0.979	,	1.874	)	0.067	0.201
							2018-2019	1.432	(	1.031	,	1.988	)	0.032	0.096

* Bonferroni corrected

[a] Period > 1 week represented by the mean of the model coefficients within the period

[b] RRR-ratio of rate ratios for prevalence/incidence outcomes; ROR-ratio of odds ratio for proportion outcomes
