# Supplementary material for: Healthcare contacts with self-harm during COVID-19: An e-cohort whole-population-based study using individual-level linked routine electronic health records in Wales, UK, 2016—March 2021
Source: PLoS One. 2022 Apr 27;17(4):e0266967. doi: 10.1371/journal.pone.0266967 (PMC9045644; doi:10.1371/journal.pone.0266967)
Supplement: S7 Table — Summary of RORs and RRRs for change in gradient of self-harm contacts by WIMD quintile for all settings (Any), primary care (GP), emergency departments (ED) and hospital admissions (HA), as well as for ED presentations with subsequent hospitalisation (ED to HA). (PDF) [file pone.0266967.s021.pdf]

Healthcare contacts with self-harm during COVID-19: an e-cohort whole-population-based study using individual-level linked routine electronic health records in Wales, UK, 2016 – March 2021

Marcos DelPozo-Banos, Sze Chim Lee, Yasmin Friedmann, Ashley Akbari, Fatemeh Torabi, Keith Lloyd, Ronan A Lyons, Ann John

S7 Table. RORs/RRRs of healthcare service contacts with self-harm in any and each setting stratified by WIMD deprivation level. Summary of RORs and RRRs for change in gradient of self-harm contacts by WIMD quintile for all settings (Any), primary care (GP), emergency departments (ED) and hospital admissions (HA), as well as for ED presentations with subsequent hospitalisation (ED to HA).

Setting	Outcome	Reference period[a]		Target period[a]		Year as counterfactual	RRR/ROR[b]	95% CI	*p*-value	*p*-value*
Any	numbers	week 1-10	30/12/2019	week 12-14	16/03/2020	2016-2017	0.986	(0.900 , 1.081)	0.770	>0.999
			to		to	2017-2018	0.903	(0.823 , 0.990)	0.029	0.087
			08/03/2020		05/04/2020	2018-2019	0.979	(0.892 , 1.076)	0.663	>0.999
Any	proportion	week 1-10	30/12/2019	week 12-14	16/03/2020	2016-2017	0.993	(0.913 , 1.080)	0.864	>0.999
			to		to	2017-2018	0.913	(0.839 , 0.994)	0.035	0.105
			08/03/2020		05/04/2020	2018-2019	0.987	(0.904 , 1.076)	0.761	>0.999
Any	numbers	week 1-10	30/12/2019	week 30-33	20/07/2020	2016-2017	1.077	(1.000 , 1.161)	0.052	0.155
			to		to	2017-2018	1.029	(0.955 , 1.108)	0.456	>0.999
			08/03/2020		16/08/2020	2018-2019	1.093	(1.015 , 1.177)	0.019	0.056
Any	proportion	week 1-10	30/12/2019	week 30-33	20/07/2020	2016-2017	1.074	(1.002 , 1.151)	0.043	0.129
			to		to	2017-2018	1.039	(0.970 , 1.112)	0.277	0.832
			08/03/2020		16/08/2020	2018-2019	1.100	(1.027 , 1.178)	0.006	**0.019**
Any	numbers	week 1-10	30/12/2019	week 50-53	07/12/2020	2016-2017	1.027	(0.945 , 1.117)	0.530	>0.999
			to		to	2017-2018	1.018	(0.938 , 1.105)	0.668	>0.999
			08/03/2020		03/01/2021	2018-2019	1.002	(0.922 , 1.089)	0.968	>0.999
Any	proportion	week 1-10	30/12/2019	week 50-53	07/12/2020	2016-2017	1.026	(0.953 , 1.104)	0.500	>0.999
			to		to	2017-2018	1.038	(0.965 , 1.116)	0.319	0.957
			08/03/2020		03/01/2021	2018-2019	0.995	(0.923 , 1.072)	0.887	>0.999
GP	numbers	week 1-10	30/12/2019	week 12-14	16/03/2020	2016-2017	0.837	(0.714 , 0.982)	0.029	0.086
			to		to	2017-2018	0.860	(0.734 , 1.008)	0.063	0.188
			08/03/2020		05/04/2020	2018-2019	0.937	(0.797 , 1.102)	0.435	>0.999
GP	proportion	week 1-10	30/12/2019	week 12-14	16/03/2020	2016-2017	0.853	(0.729 , 0.997)	0.046	0.139
			to		to	2017-2018	0.882	(0.755 , 1.030)	0.114	0.341
			08/03/2020		05/04/2020	2018-2019	0.952	(0.812 , 1.116)	0.545	>0.999
GP	numbers	week 1-10	30/12/2019	week 30-33	20/07/2020	2016-2017	1.037	(0.907 , 1.185)	0.597	>0.999
			to		to	2017-2018	1.065	(0.932 , 1.218)	0.356	>0.999
			08/03/2020		16/08/2020	2018-2019	1.019	(0.893 , 1.163)	0.779	>0.999
GP	proportion	week 1-10	30/12/2019	week 30-33	20/07/2020	2016-2017	1.059	(0.928 , 1.207)	0.396	>0.999
			to		to	2017-2018	1.071	(0.939 , 1.222)	0.307	0.922

			08/03/2020		16/08/2020	2018-2019	1.035 (0.909 , 1.179)	0.600	>0.999
GP	numbers	week 1-10	30/12/2019	week 50-53	07/12/2020	2016-2017	1.021 (0.880 , 1.186)	0.782	>0.999
			to		to	2017-2018	1.038 (0.898 , 1.200)	0.614	>0.999
			08/03/2020		03/01/2021	2018-2019	0.939 (0.808 , 1.092)	0.415	>0.999
GP	proportion	week 1-10	30/12/2019	week 50-53	07/12/2020	2016-2017	1.007 (0.875 , 1.160)	0.919	>0.999
			to		to	2017-2018	1.046 (0.910 , 1.202)	0.531	>0.999
			08/03/2020		03/01/2021	2018-2019	0.950 (0.823 , 1.097)	0.484	>0.999
ED	numbers	week 1-10	30/12/2019	week 12-14	16/03/2020	2016-2017	1.107 (0.994 , 1.234)	0.064	0.192
			to		to	2017-2018	0.953 (0.854 , 1.063)	0.388	>0.999
			08/03/2020		05/04/2020	2018-2019	1.046 (0.936 , 1.169)	0.425	>0.999
ED	proportion	week 1-10	30/12/2019	week 16-17	13/04/2020	2016-2017	1.014 (0.889 , 1.156)	0.841	>0.999
			to		to	2017-2018	1.054 (0.924 , 1.202)	0.435	>0.999
			08/03/2020		26/04/2020	2018-2019	0.995 (0.873 , 1.135)	0.946	>0.999
ED	numbers	week 1-10	30/12/2019	week 30-33	20/07/2020	2016-2017	1.127 (1.036 , 1.227)	0.005	**0.016**
			to		to	2017-2018	0.983 (0.905 , 1.068)	0.680	>0.999
			08/03/2020		16/08/2020	2018-2019	1.090 (1.004 , 1.184)	0.040	0.121
ED	proportion	week 1-10	30/12/2019	week 41-45	05/10/2020	2016-2017	1.079 (0.987 , 1.179)	0.094	0.282
			to		to	2017-2018	0.948 (0.868 , 1.035)	0.230	0.689
			08/03/2020		08/11/2020	2018-2019	1.041 (0.954 , 1.136)	0.370	>0.999
ED	numbers	week 1-10	30/12/2019	week 50-53	07/12/2020	2016-2017	1.080 (0.981 , 1.189)	0.115	0.346
			to		to	2017-2018	1.041 (0.947 , 1.144)	0.405	>0.999
			08/03/2020		03/01/2021	2018-2019	0.990 (0.899 , 1.090)	0.836	>0.999
ED	proportion	week 1-10	30/12/2019	week 53-58	28/12/2020	2016-2017	1.123 (1.035 , 1.218)	0.005	**0.015**
			to		to	2017-2018	0.988 (0.910 , 1.072)	0.768	>0.999
			08/03/2020		07/02/2021	2018-2019	1.058 (0.976 , 1.147)	0.174	0.521
ED to HA	numbers	week 1-10	30/12/2019	week 12-14	16/03/2020	2016-2017	1.103 (0.891 , 1.364)	0.368	>0.999
			to		to	2017-2018	0.993 (0.801 , 1.229)	0.946	>0.999
			08/03/2020		05/04/2020	2018-2019	1.079 (0.867 , 1.345)	0.495	>0.999
ED to HA	proportion	week 1-10	30/12/2019	week 19-24	04/05/2020	2016-2017	1.017 (0.908 , 1.139)	0.769	>0.999
			to		to	2017-2018	0.953 (0.852 , 1.067)	0.406	>0.999
			08/03/2020		14/06/2020	2018-2019	0.997 (0.895 , 1.110)	0.956	>0.999
ED to HA	numbers	week 1-10	30/12/2019	week 30-33	20/07/2020	2016-2017	1.153 (0.974 , 1.364)	0.099	0.297
			to		to	2017-2018	0.996 (0.847 , 1.172)	0.961	>0.999
			08/03/2020		16/08/2020	2018-2019	1.072 (0.911 , 1.262)	0.401	>0.999
ED to HA	proportion	week 1-10	30/12/2019	week 36-38	31/08/2020	2016-2017	1.002 (0.850 , 1.182)	0.979	>0.999
			to		to	2017-2018	0.911 (0.768 , 1.079)	0.280	0.841
			08/03/2020		20/09/2020	2018-2019	0.842 (0.708 , 1.000)	0.050	0.149
ED to HA	numbers	week 1-10	30/12/2019	week 50-53	07/12/2020	2016-2017	1.091 (0.890 , 1.336)	0.402	>0.999
			to		to	2017-2018	0.986 (0.808 , 1.203)	0.891	>0.999

			08/03/2020		03/01/2021	2018-2019	0.895 (0.730 , 1.096)	0.282	0.846
ED to HA	proportion	week 1-10	30/12/2019	week 47-60	16/11/2020	2016-2017	1.038 (0.935 , 1.153)	0.483	>0.999
			to		to	2017-2018	0.947 (0.855 , 1.048)	0.295	0.884
			08/03/2020		21/02/2021	2018-2019	0.981 (0.887 , 1.085)	0.705	>0.999
HA	numbers	week 1-10	30/12/2019	week 12-14	16/03/2020	2016-2017	0.931 (0.803 , 1.079)	0.343	>0.999
			to		to	2017-2018	0.865 (0.746 , 1.004)	0.056	0.169
			08/03/2020		05/04/2020	2018-2019	0.944 (0.807 , 1.104)	0.472	>0.999
HA	proportion	week 1-10	30/12/2019	week 15-33	06/04/2020	2016-2017	0.993 (0.918 , 1.075)	0.863	>0.999
			to		to	2017-2018	0.951 (0.879 , 1.028)	0.207	0.620
			08/03/2020		16/08/2020	2018-2019	0.999 (0.921 , 1.085)	0.988	>0.999
HA	numbers	week 1-10	30/12/2019	week 30-33	20/07/2020	2016-2017	1.051 (0.927 , 1.191)	0.439	>0.999
			to		to	2017-2018	1.038 (0.916 , 1.175)	0.560	>0.999
			08/03/2020		16/08/2020	2018-2019	1.162 (1.025 , 1.316)	0.019	0.056
HA	proportion	week 1-10	30/12/2019	week 41-44	05/10/2020	2016-2017	0.963 (0.852 , 1.090)	0.552	>0.999
			to		to	2017-2018	0.851 (0.751 , 0.964)	0.011	**0.033**
			08/03/2020		01/11/2020	2018-2019	1.001 (0.884 , 1.132)	0.993	>0.999
HA	numbers	week 1-10	30/12/2019	week 30-33	20/07/2020	2016-2017	0.984 (0.846 , 1.144)	0.832	>0.999
			to		to	2017-2018	0.941 (0.813 , 1.090)	0.419	>0.999
			08/03/2020		16/08/2020	2018-2019	0.960 (0.827 , 1.114)	0.590	>0.999
HA	proportion	week 1-10	30/12/2019	week 50-53	07/12/2020	2016-2017	0.975 (0.848 , 1.122)	0.725	>0.999
			to		to	2017-2018	0.939 (0.819 , 1.077)	0.367	>0.999
			08/03/2020		03/01/2021	2018-2019	0.956 (0.832 , 1.099)	0.527	>0.999

* Bonferroni corrected
[a] Period > 1 week represented by the mean of the model coefficients within the period
[b] RRR-ratio of rate ratios for prevalence/incidence outcomes; ROR-ratio of odds ratio for proportion outcomes
