# Supplementary material for: Healthcare contacts with self-harm during COVID-19: An e-cohort whole-population-based study using individual-level linked routine electronic health records in Wales, UK, 2016—March 2021
Source: PLoS One. 2022 Apr 27;17(4):e0266967. doi: 10.1371/journal.pone.0266967 (PMC9045644; doi:10.1371/journal.pone.0266967)
Supplement: S8 Table — Summary of RORs comparing change in proportion of people who self-harmed and were in contract with primary care (GP), emergency departments (ED) and/or hospital admissions (HA) between reference and target periods to the respective changes in previous years stratified by WIMD deprivation level. (PDF) [file pone.0266967.s022.pdf]

Healthcare contacts with self-harm during COVID-19: an e-cohort whole-population-based study using individual-level linked routine electronic health records in Wales, UK, 2016 – March 2021

Marcos DelPozo-Banos, Sze Chim Lee, Yasmin Friedmann, Ashley Akbari, Fatemeh Torabi, Keith Lloyd, Ronan A Lyons, Ann John

S8 Table. RORs of people in contact with one or more healthcare settings with self-harm stratified by WIMD deprivation level. Summary of RORs comparing change in proportion of people who self-harmed and were in contract with primary care (GP), emergency departments (ED) and/or hospital admissions (HA) between reference and target periods to the respective changes in previous years stratified by WIMD deprivation level.

Setting	Reference period[a]		Target period[a]		Year as counterfactual	RRR/ROR[b]	95% CI		*p*-value	*p*-value*
GP	week 1-10	30/12/2019	week 14-18	30/03/2020	2016-2017	0.921	(0.835 ,	1.016)	0.101	0.303
		to		to	2017-2018	0.924	(0.835 ,	1.022)	0.123	0.369
		08/03/2020		03/05/2020	2018-2019	0.957	(0.866 ,	1.058)	0.390	>0.999
GP	week 1-10	30/12/2019	week 28-33	06/07/2020	2016-2017	0.942	(0.857 ,	1.037)	0.222	0.667
		to		to	2017-2018	0.999	(0.908 ,	1.098)	0.977	>0.999
		08/03/2020		16/08/2020	2018-2019	0.957	(0.871 ,	1.052)	0.363	>0.999
ED	week 1-10	30/12/2019	week 12-15	16/03/2020	2016-2017	1.094	(1.015 ,	1.179)	0.018	0.055
		to		to	2017-2018	1.053	(0.977 ,	1.136)	0.177	0.532
		08/03/2020		12/04/2020	2018-2019	1.034	(0.958 ,	1.116)	0.396	>0.999
ED	week 1-10	30/12/2019	week 28-33	06/07/2020	2016-2017	1.045	(0.989 ,	1.104)	0.121	0.362
		to		to	2017-2018	0.990	(0.938 ,	1.044)	0.711	>0.999
		08/03/2020		16/08/2020	2018-2019	1.020	(0.966 ,	1.077)	0.472	>0.999
HA	week 1-10	30/12/2019	week 12-13	16/03/2020	2016-2017	0.926	(0.814 ,	1.054)	0.243	0.729
		to		to	2017-2018	0.989	(0.873 ,	1.121)	0.868	>0.999
		08/03/2020		29/03/2020	2018-2019	0.982	(0.848 ,	1.136)	0.803	>0.999
HA	week 1-10	30/12/2019	week 28-33	06/07/2020	2016-2017	0.981	(0.899 ,	1.070)	0.658	>0.999
		to		to	2017-2018	1.000	(0.918 ,	1.089)	0.998	>0.999
		08/03/2020		16/08/2020	2018-2019	1.016	(0.929 ,	1.111)	0.730	>0.999
HA	week 1-10	30/12/2019	week 58-61	01/02/2021	2016-2017	1.014	(0.906 ,	1.135)	0.807	>0.999
		to		to	2017-2018	0.963	(0.855 ,	1.085)	0.536	>0.999
		08/03/2020		28/02/2021	2018-2019	1.005	(0.897 ,	1.126)	0.933	>0.999
GP only	week 1-10	30/12/2019	week 14-18	30/03/2020	2016-2017	0.898	(0.785 ,	1.027)	0.117	0.350
		to		to	2017-2018	0.958	(0.833 ,	1.100)	0.540	>0.999
		08/03/2020		03/05/2020	2018-2019	0.963	(0.841 ,	1.103)	0.587	>0.999
GP only	week 1-10	30/12/2019	week 28-33	06/07/2020	2016-2017	0.882	(0.772 ,	1.008)	0.065	0.194
		to		to	2017-2018	0.960	(0.840 ,	1.097)	0.546	>0.999
		08/03/2020		16/08/2020	2018-2019	0.893	(0.784 ,	1.017)	0.087	0.262

ED only	week 1-10	30/12/2019	week 12-15	16/03/2020	2016-2017	1.120	(	1.006	,	1.246	)	0.039	0.116
		to		to	2017-2018	1.062	(	0.953	,	1.184	)	0.277	0.831
		08/03/2020		12/04/2020	2018-2019	1.019	(	0.914	,	1.137	)	0.732	>0.999
ED only	week 1-10	30/12/2019	week 28-33	06/07/2020	2016-2017	1.041	(	0.962	,	1.127	)	0.321	0.963
		to		to	2017-2018	1.004	(	0.929	,	1.086	)	0.914	>0.999
		08/03/2020		16/08/2020	2018-2019	1.028	(	0.951	,	1.112	)	0.489	>0.999
ED only	week 1-10	30/12/2019	week 50-53	07/12/2020	2016-2017	1.017	(	0.927	,	1.116	)	0.723	>0.999
		to		to	2017-2018	1.048	(	0.957	,	1.148	)	0.315	0.944
		08/03/2020		03/01/2021	2018-2019	1.036	(	0.944	,	1.137	)	0.455	>0.999
ED only	week 1-10	30/12/2019	week 58-61	01/02/2021	2016-2017	1.027	(	0.942	,	1.120	)	0.541	>0.999
		to		to	2017-2018	1.032	(	0.944	,	1.129	)	0.488	>0.999
		08/03/2020		28/02/2021	2018-2019	1.012	(	0.923	,	1.110	)	0.796	>0.999
HA only	week 1-10	30/12/2019	week 14-18	30/03/2020	2016-2017	0.959	(	0.775	,	1.186	)	0.697	>0.999
		to		to	2017-2018	0.983	(	0.797	,	1.212	)	0.873	>0.999
		08/03/2020		03/05/2020	2018-2019	0.948	(	0.752	,	1.196	)	0.654	>0.999
HA only	week 1-10	30/12/2019	week 28-33	06/07/2020	2016-2017	0.998	(	0.862	,	1.156	)	0.978	>0.999
		to		to	2017-2018	1.063	(	0.918	,	1.232	)	0.415	>0.999
		08/03/2020		16/08/2020	2018-2019	1.026	(	0.882	,	1.194	)	0.736	>0.999
GP & ED only	week 1-10	30/12/2019	week 14-18	30/03/2020	2016-2017	1.607	(	0.990	,	2.608	)	0.055	0.165
		to		to	2017-2018	1.134	(	0.717	,	1.795	)	0.591	>0.999
		08/03/2020		03/05/2020	2018-2019	1.322	(	0.822	,	2.125	)	0.250	0.749
GP & ED only	week 1-10	30/12/2019	week 27-30	29/06/2020	2016-2017	1.423	(	0.824	,	2.459	)	0.206	0.617
		to		to	2017-2018	1.490	(	0.866	,	2.563	)	0.150	0.449
		08/03/2020		26/07/2020	2018-2019	1.116	(	0.662	,	1.881	)	0.680	>0.999
GP & ED only	week 1-10	30/12/2019	week 35-36	24/08/2020	2016-2017	0.978	(	0.619	,	1.546	)	0.924	>0.999
		to		to	2017-2018	1.011	(	0.626	,	1.634	)	0.963	>0.999
		08/03/2020		06/09/2020	2018-2019	0.941	(	0.580	,	1.525	)	0.804	>0.999
GP & HA only	week 1-10	30/12/2019	week 14-18	30/03/2020	2016-2017	0.776	(	0.553	,	1.088	)	0.141	0.424
		to		to	2017-2018	0.706	(	0.504	,	0.991	)	0.044	0.132
		08/03/2020		03/05/2020	2018-2019	0.961	(	0.666	,	1.386	)	0.829	>0.999
GP & HA only	week 1-10	30/12/2019	week 28-33	06/07/2020	2016-2017	0.823	(	0.603	,	1.123	)	0.218	0.655
		to		to	2017-2018	0.961	(	0.708	,	1.304	)	0.798	>0.999
		08/03/2020		16/08/2020	2018-2019	1.070	(	0.771	,	1.484	)	0.687	>0.999
ED & HA only	week 1-10	30/12/2019	week 12-15	16/03/2020	2016-2017	1.264	(	0.998	,	1.601	)	0.052	0.156
		to		to	2017-2018	1.104	(	0.871	,	1.400	)	0.413	>0.999
		08/03/2020		12/04/2020	2018-2019	1.170	(	0.923	,	1.484	)	0.194	0.583

ED & HA only	week 1-10	30/12/2019	week 28-33	06/07/2020	2016-2017	0.984	(0.809 , 1.197)	0.872	>0.999
		to		to	2017-2018	0.890	(0.742 , 1.069)	0.212	0.637
		08/03/2020		16/08/2020	2018-2019	0.973	(0.802 , 1.179)	0.777	>0.999
ED & HA only	week 1-10	30/12/2019	week 58-61	01/02/2021	2016-2017	1.314	(1.011 , 1.707)	0.041	0.124
		to		to	2017-2018	0.940	(0.712 , 1.241)	0.664	>0.999
		08/03/2020		28/02/2021	2018-2019	1.251	(0.964 , 1.624)	0.092	0.275
GP, ED & HA only	week 1-10	30/12/2019	week 14-18	30/03/2020	2016-2017	0.893	(0.592 , 1.348)	0.591	>0.999
		to		to	2017-2018	0.967	(0.620 , 1.507)	0.881	>0.999
		08/03/2020		03/05/2020	2018-2019	0.961	(0.632 , 1.461)	0.852	>0.999
GP, ED & HA only	week 1-10	30/12/2019	week 28-33	06/07/2020	2016-2017	1.030	(0.694 , 1.527)	0.885	>0.999
		to		to	2017-2018	1.001	(0.680 , 1.473)	0.997	>0.999
		08/03/2020		16/08/2020	2018-2019	1.194	(0.797 , 1.789)	0.391	>0.999
GP, ED & HA only	week 1-10	30/12/2019	week 58-61	01/02/2021	2016-2017	1.152	(0.667 , 1.989)	0.612	>0.999
		to		to	2017-2018	1.044	(0.607 , 1.794)	0.877	>0.999
		08/03/2020		28/02/2021	2018-2019	0.953	(0.532 , 1.706)	0.870	>0.999

* Bonferroni corrected

[a] Period > 1 week represented by the mean of the model coefficients within the period

[b] RRR-ratio of rate ratios for prevalence/incidence outcomes; ROR-ratio of odds ratio for proportion outcomes
